# Urbanization and a green corridor do not impact genetic divergence in common milkweed (*Asclepias syriaca* L.)

**DOI:** 10.1038/s41598-023-47524-8

**Published:** 2023-11-22

**Authors:** Sophie T. Breitbart, Anurag A. Agrawal, Helene H. Wagner, Marc T. J. Johnson

**Affiliations:** 1 Department of Ecology and Evolutionary Biology, University of Toronto, 25 Willcocks Street, Toronto, ON M5S 3B2 Canada; 2 Department of Biology, University of Toronto Mississauga, 3359 Mississauga Road, Mississauga, ON L5L 1C6 Canada; 3 Centre for Urban Environments, University of Toronto Mississauga, 3359 Mississauga Road, Mississauga, ON L5L 1C6 Canada; 4 Department of Ecology and Evolutionary Biology, Cornell University, E145 Corson Hall, Ithaca, NY 14853 USA; 5 Department of Entomology, Cornell University, 2126 Comstock Hall, Ithaca, NY 14853 USA

**Keywords:** Ecological genetics, Evolution

## Abstract

Urbanization is altering landscapes globally at an unprecedented rate. While ecological differences between urban and rural environments often promote phenotypic divergence among populations, it is unclear to what degree these trait differences arise from genetic divergence as opposed to phenotypic plasticity. Furthermore, little is known about how specific landscape elements, such as green corridors, impact genetic divergence in urban environments. We tested the hypotheses that: (1) urbanization, and (2) proximity to an urban green corridor influence genetic divergence in common milkweed (*Asclepias syriaca*) populations for phenotypic traits. Using seeds from 52 populations along three urban-to-rural subtransects in the Greater Toronto Area, Canada, one of which followed a green corridor, we grew ~ 1000 plants in a common garden setup and measured > 20 ecologically-important traits associated with plant defense/damage, reproduction, and growth over four years. We found significant heritable variation for nine traits within common milkweed populations and weak phenotypic divergence among populations. However, neither urbanization nor an urban green corridor influenced genetic divergence in individual traits or multivariate phenotype. These findings contrast with the expanding literature demonstrating that urbanization promotes rapid evolutionary change and offer preliminary insights into the eco-evolutionary role of green corridors in urban environments.

## Introduction

The intense environmental changes associated with urbanization can profoundly alter the ecological processes of urban-dwelling populations. Several biotic and abiotic factors differentiate urban from non-urban environments including higher pollution, habitat fragmentation, and temperature due to the urban heat island effect^1,2^. In turn, these alterations can impact populations and communities directly by affecting species richness^3,4^, abundance^5,6^, and spatiotemporal distribution^7,8^. These environmental conditions can also impose indirect impacts by restructuring species interactions such as antagonisms (e.g., predator/prey, including herbivory) and mutualisms (e.g., plant-pollinator and plant–microbe)^9–11^. In response, individuals and ultimately populations have experienced trait changes associated with growth, reproduction, defense/damage, morphology**,** and behavior, a process indicative of intraspecific phenotypic divergence^12–16^. Despite the recent rise of studies documenting phenotypic divergence among urban and rural populations^12,13,15^, few have distinguished the degree to which evolution and phenotypic plasticity drive these patterns.

Phenotypic divergence can be explained by two general mechanisms: phenotypic plasticity and genetic divergence. Phenotypic plasticity occurs when individuals with identical genotypes exhibit different phenotypes in different environments^17^ and was shown to account for the majority of the increased heat tolerance exhibited by urban water fleas (*Daphnia magna*) in a common garden experiment^18^. Conversely, genetic divergence happens via evolution and is, in urban environments, most commonly attributed to changes in natural selection, genetic drift, and gene flow^19–21^. Specifically, the widespread habitat fragmentation characteristic of urban environments is frequently implicated in reports of heightened genetic drift within populations^22,23^, restricted gene flow among populations^24,25^, and natural selection^26^.

Several studies have investigated the basis of phenotypic divergence in plants^27–30^, yet few included multiple traits, or traits from diverse ecological functions such as reproduction, defense/damage, and growth^16,29,30^. Thus, we have a poor understanding of how urbanization affects divergence in the multivariate phenotype. Individual responses to environmental change often involve multiple correlated traits^31,32^, and excluding traits from major categories (e.g., defense) complicates the detection of shifts in life history strategies, including trade-offs, or the lack thereof. Thus, surveying a broad range of phenotypic traits is essential for determining the drivers of phenotypic divergence in urban environments.

Individual elements of the urban landscape are thought to impact the evolution of phenotypic traits. Long, connected habitat patches—henceforth, green corridors— have been shown to impact evolution in non-urban areas^33–35^ such as forest fragments, frequently by facilitating gene flow^36,37^ and alleviating effects of genetic drift^38^ (but see Orrock^39^). In turn, these processes can accelerate adaptation through the introduction of beneficial alleles or slow it through the introduction of deleterious or neutral alleles^40–43^. However, virtually nothing is known about how these corridors impact phenotypic trait evolution in urban areas. Broadly, urban green corridors frequently impact dispersal and gene flow^44,45^, such as in the white-footed mouse (*Peromyscus leucopus*) in New York where vegetation corridors facilitate dispersal^46^. Investigating how urban green corridors influence genetic divergence in phenotypic traits is essential for discerning whether aspects of urban landscapes shape the phenotype of urban-dwelling taxa. This knowledge would also offer conservation agencies important context about the evolutionary consequences of corridors primarily utilized to remedy the ecological costs of habitat fragmentation in urban environments^47^. Evaluating how urban green corridors impact the evolution of phenotypic traits represents an important step towards understanding evolutionary dynamics in heterogeneous urban landscapes.

Prior research demonstrating how urbanization influences ecological change in a native plant of conservation importance, common milkweed (*Asclepias syriaca*)*,* as well as its pollinators and herbivores, suggests that urbanization may be a powerful agent of natural selection in this system^48,49^. In an observational survey, urbanization influenced reproductive success and pollinator community structure, while in urban populations, proximity to a green corridor inconsistently affected reproductive success, but uniformly decreased pollinator diversity and richness^49^. Likewise, another study highlighted the complexity with which nine specialist herbivore species were impacted by urbanization and season across six cities^48^. Urban common milkweed populations showed higher herbivore species richness yet less leaf herbivory than rural populations, with these effects varying by season and city. Additionally, the milkweed leaf-mining fly (*Liriomyza asclepiadis*) was 90% more abundant in urban areas and the milkweed stem weevil (*Rhyssomatus lineaticollis*) was 41% more abundant in rural areas, although the effect for the milkweed stem weevil varied by city. Consequently, differential ecological stressors within urban areas could drive the evolution of defense and reproductive traits in common milkweed.

Here, we used a common garden experiment to test the hypotheses that: (1) urbanization, and (2) proximity to an urban green corridor drive genetic divergence in phenotypic traits among populations of common milkweed. We asked three questions: (Q1) Is there heritable variation for phenotypic traits within populations and genetic divergence between populations? These are two necessary conditions if common milkweed is to evolve in response to urban environmental change^17,50^. (Q2) To what extent is urbanization associated with genetic divergence in phenotypic traits among populations? If common milkweed has experienced genetic divergence in response to urbanization, then population-level phenotypic trait estimates will correlate with local urbanization levels. (Q3) Is proximity to an urban green corridor related to the levels of genetic divergence among urban populations? If so, there will be distinct differences in phenotypic trait estimates between populations near to and far from the corridor. This study builds upon work investigating how urbanization and a green corridor influence phenotypic divergence in reproduction in common milkweed^49^ by uncovering the extent to which evolution and phenotypic plasticity shape phenotypic divergence in reproductive, growth, and defense-related traits. These results provide insights into how plants are likely to respond to rapid environmental change in heterogeneous urban environments.

## Methods

### Study system

Common milkweed is an herbaceous perennial plant native to eastern North America. Although common milkweed grows in discrete patches of one to thousands of ramets (stems), often in abandoned agricultural fields^51^, urban populations tend to be smaller and inhabit public parks, railway and transmission rights-of-way, and roadsides, as well as private lawns and gardens. Plants can reproduce vegetatively through rhizomes that generate clonal ramets, or sexually through the cross-pollination of hermaphroditic flowers, each with five pollen sac pairs collectively called pollinaria^51–53^. Fertilization following successful pollination by insects, such as the western honey bee (*Apis mellifera*), bumblebees (*Bombus* spp*.*), and *Halictidae* spp. (*Hymenoptera*) in urban areas^49,54,55^, yields follicles (i.e., fruits) filled with wind-dispersed seeds^52,56^. Common milkweed is also recognized for its conservation significance because milkweeds (*Asclepias* spp.) provide essential resources for the threatened Monarch butterfly (*Danaus plexippus*)^57^.

Multiple traits protect common milkweed against herbivores. Milkweeds contain a pressurized, milky sap called latex that physically interferes with herbivores chewing tissue by gumming their mouthparts^58–60^. Herbivores that can overcome this barrier must also tolerate cardenolides, a suite of secondary metabolites which can disrupt sodium–potassium pumps (Na^+^/K^+^-ATPases) required for maintaining membrane potential^61^. At least 16 herbivores have coevolved with milkweeds and developed tolerance to these defenses^61,62^, and at least nine, including the Monarch butterfly, can enhance their own toxicities by sequestering cardenolides^62,63^. Compared to growth and reproduction, heritabilities of defense traits (e.g., latex and cardenolides) are often high^64–68^, indicating an increased likelihood for traits in this category to evolve.

### Field sampling of maternal families

**Figure 1:**
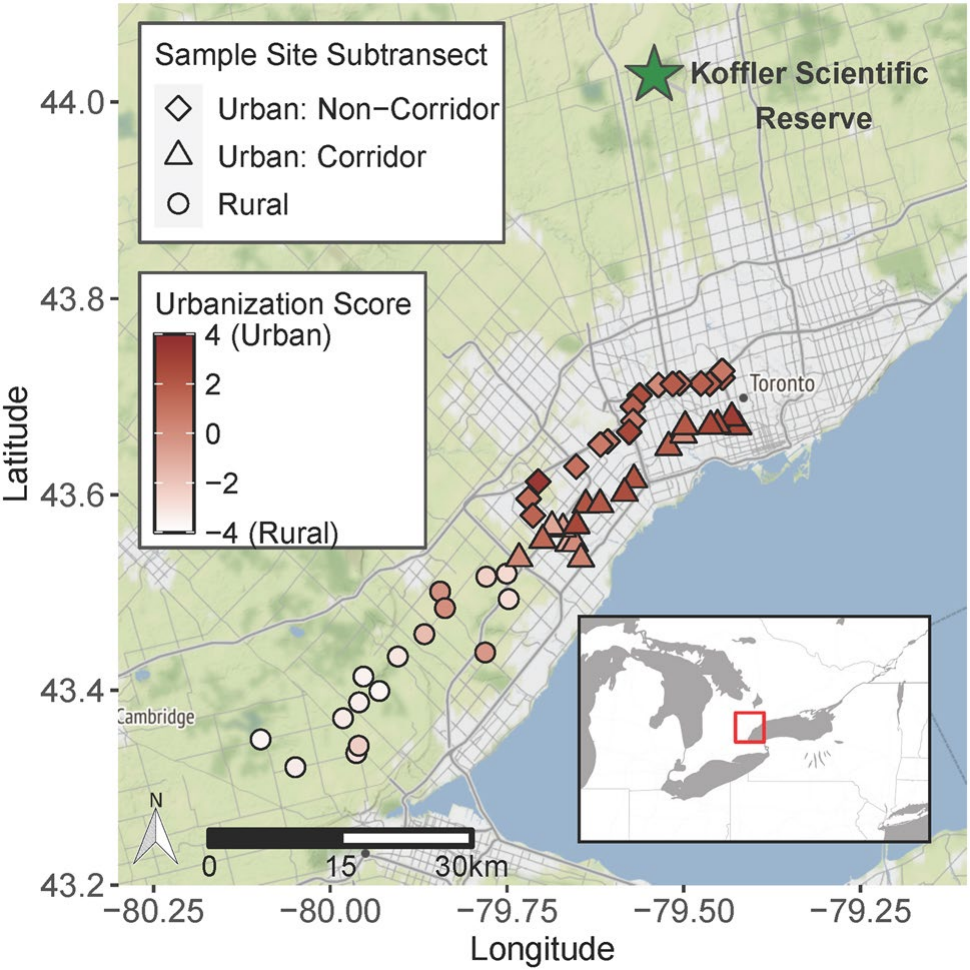
Map of 52 common milkweed populations sampled along Toronto’s urban–rural gradient and location of common garden experiment at the Koffler Scientific Reserve (green star). Urban: Non-Corridor populations (squares; N = 17). Urban: Corridor populations (triangles; N = 19). Rural populations (circles; N = 16). The color of the symbols indicates urbanization score, where positive values indicate a high degree of urbanization (based on the quantity of vegetation, buildings, and paved roads per 1 km^2^). The Stamen terrain basemap shows urban and suburban areas in light gray, nonurban agricultural and forested areas in green, and Lake Ontario in blue. Map tiles by Stamen Design, under CC BY 3.0. Data by OpenStreetMap, under ODbL.

To evaluate whether urbanization affects the evolution of ecologically important phenotypic traits, we sampled plants at 52 sites along a 67 km urban–rural gradient in the Greater Toronto Area, Ontario, Canada (Fig. 1) as described in Breitbart et al.^49^. This gradient was split into two parallel urban subtransects ca. 5 km apart, and a third rural subtransect, all approximately the same length (30–35 km). The northern urban subtransect (“Urban: Non-Corridor”, N = 17 sites) crossed through the urban matrix while the southern urban subtransect (“Urban: Corridor”, N = 19 sites) followed a green corridor: a mostly continuous vegetated strip of land, ca. 50–350 m wide, that ran adjacent to linear features like trails, rights-of-way, and railways. Sampling sites were located within or near the corridor. The rural subtransect (“Rural”, N = 16 sites) extended westward from the suburban termini of the urban subtransects. Sampling sites (henceforth “populations”) of isolated patches of common milkweed were spaced > 500 m apart. We then divided the population into five equally-sized sections and collected seeds from one follicle (i.e., full-sibling seed family) per ramet per section, aiming to separate ramets by > 3 m to avoid resampling the same clone. Seeds were stored in small paper (coin) envelopes and preserved at − 20 °C until germination. We performed the seed collection in accordance with the relevant guidelines and regulations (e.g., obtaining permission before sampling on private land).

### Urbanization metrics

We quantified urbanization using two methods as described in Breitbart et al.^49^. Briefly, we first measured the distance from each population to the Toronto urban center (43.6563, − 79.3809), as a proxy for the degree of urbanization. Distance from the urban center is correlated with numerous environmental factors associated with urbanization^69^, and, for this city specifically, has been shown to influence the ecology and evolution of other plant, herbivore, and pollinator communities^10,70,71^. We also calculated an urbanization score for each population with the UrbanizationScore software^72–74^, which ranged from − 3.56 (least urban) to 3.37 (most urban). For each population, the software downloaded a 1 km radius aerial at 100 m resolution from Google Maps (Google LLC, Mountain View, California). The amount of vegetation, buildings, and roads was quantified and used to calculate landscape-cover variables, which were finally combined with principal component analysis into an urbanization score. Urbanization metrics were highly correlated (F_(1,49)_ = 122.952, *p* < 0.001, R^2^_adj_ = 0.709) (Supplementary Fig. S1).

### Common garden experiment

To test if urbanization and an urban green corridor drive genetic divergence in phenotypic traits, we conducted a common garden experiment in 2019–2022. We germinated ~ 10 full-sibling seeds from each of 5 families per population, then grew seeds in pots in a growth chamber for six weeks. Seedlings were then snipped at the base of the ramet to ensure equal baseline heights and transported to the University of Toronto’s Koffler Scientific Reserve (44.029, − 79.531) in King City, Ontario, Canada (http://ksr.utoronto.ca/) in May 2019. Seedlings were transplanted into 3.79 L circular pots filled with field soil and topped with 2.5 cm Triple Mix, then randomized into 4 contiguous spatial blocks with 1.5 m spacing between rows and columns (Supplementary Fig. S2). Each row was covered with a 1 m-wide strip of landscaping fabric (Quest Better Barriers, Brampton, Canada) to prevent weeds from outcompeting experimental plants. Overall, we planted 954 individuals from 52 populations, 208 full-sib families, and 3 subtransects (mean: 4 families/population; 4.55 plants/family; 18.33 plants/population; range: 1–25 plants/population), with variation in representation due to germination success. We measured ecologically important traits (described below) related to plant growth, defense/damage, and reproduction over four years, in accordance with the relevant governmental and university guidelines and regulations.

### Trait measurements

**Figure 2:**
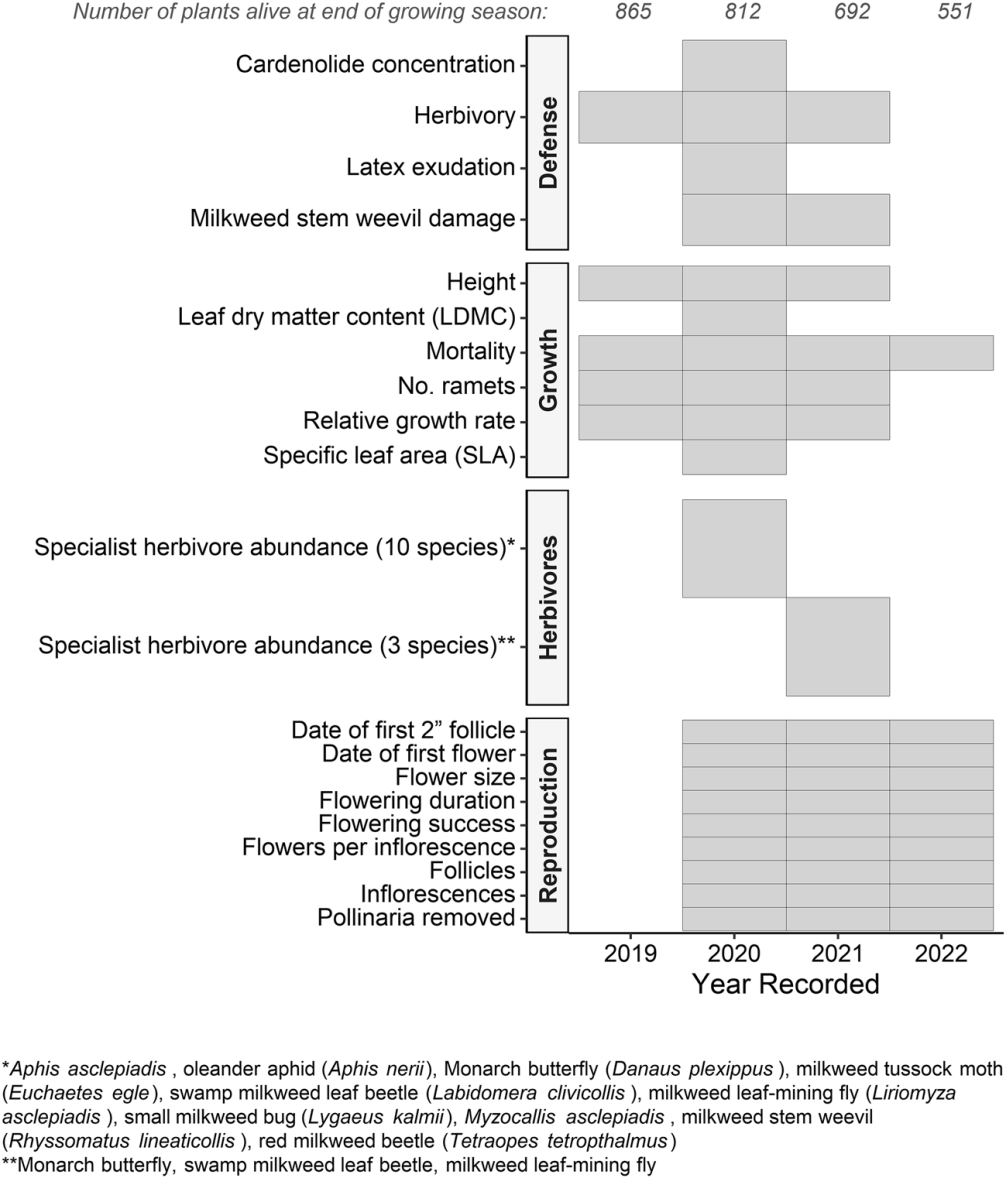
Schedule for measurement of traits associated with plant reproduction, growth, and defense/damage throughout 2019–2022 field seasons and the number of plants alive per year. Grey boxes indicate years of measurement.

In total, we measured 27 traits from 3 functional categories: plant defense/damage, growth/development, and reproduction, as well as herbivore abundance to contextualize defense trait expression (Table 1, Fig. 2). Measuring this large number of traits with diverse ecological functions allowed us to assess how the multivariate phenotype of common milkweed was evolving in response to urban environmental gradients.

**Table 1:** Broad-sense heritability (*H*^2^), coefficient of genetic variation (CV_G_), heritable genetic variation within populations (*p* (Family)), standardized measure of genetic differentiation among populations for quantitative traits (Q_ST_), and heritable genetic variation among populations (*p* (Population)) for phenotypic traits.

	*H*^2^	CV_G_	*p* (Family)	Q_ST_	*p* (Population)
**Defense/damage**					
Latex exudation	0.092	0.015	0.077	0.174	**0.016**
Herbivory before flowering (binary)	0.021	0.271	0.490	0.000	0.500
Herbivory before flowering (quantitative)	0.000	0.000	0.500	1.000	0.500
Herbivory after flowering (binary)	0.588	0.948	**0.004**	0.000	0.496
Herbivory after flowering (quantitative)	0.043	4.456	0.260	0.000	0.500
Milkweed stem weevil damage (binary)	0.418	0.995	**0.030**	0.000	0.500
Milkweed stem weevil damage (quantitative)	0.080	0.031	0.138	0.116	0.136
**Reproduction**					
Flowering success	0.102	0.309	0.302	0.492	**0.014**
Flowers per inflorescence	0.000	0.000	0.500	1.000	0.180
Flower size	0.082	0.042	0.370	0.305	0.134
Flowering duration	0.000	0.000	0.500	0.555	0.500
Date of first flower	0.000	0.010	**< 0.001**	0.000	0.500
Pollinaria removed	0.544	0.299	**0.034**	0.009	0.449
Follicles	0.000	0.000	0.500	0.287	0.500
Date of first follicle	0.235	0.034	**< 0.001**	0.002	0.500
Inflorescences	0.000	0.000	0.496	1.000	**0.035**
**Herbivore abundance**					
Monarch butterfly abundance	0.003	0.767	**0.021**	0.052	0.396
Milkweed leaf-mining fly abundance	0.055	0.140	0.142	0.162	0.106
Swamp milkweed leaf beetle abundance	0.000	0.011	0.500	0.122	0.500
**Growth**					
LDMC	0.000	0.000	0.500	0.011	0.380
SLA	0.000	0.000	0.500	1.000	0.404
Height before flowering	0.069	0.004	0.112	0.000	0.500
Height after flowering	0.132	0.008	**0.009**	0.052	0.189
Relative growth rate	0.021	3.643	0.394	0.000	0.500
Ramets before flowering	0.161	0.127	**< 0.001**	0.009	0.429
Ramets after flowering	0.138	0.189	**< 0.001**	0.017	0.364
Mortality	0.178	0.320	0.189	0.139	0.186

Significant *p*-values (*p* ≤ 0.05) are bolded.

We assessed five ecologically important traits related to plant defense/damage (Supplementary Text S1). We measured leaf herbivory by chewing herbivores before and after flowering by selecting the five oldest leaves pointing east (for consistency) and visually estimating the percent leaf area removed as described in Johnson et al.^75^. We quantified stem damage by a specialist milkweed stem weevil as the sum length of the oviposition scars per plant, which strongly predicts the number of eggs deposited into the ramet^76^. Latex exudation (the amount of latex released from plant tissue) was quantified by snipping the tip (~ 0.5 cm) off the youngest fully expanded intact leaf and collecting the latex exudate on a pre-weighed 1 cm filter paper disc as described in Agrawal et al.^64^. Discs were placed in a pre-weighed microcentrifuge tube on dry ice, then transferred to a − 80 °C freezer until weighed to the nearest 1 µg.

We generated population-level estimates of leaf cardenolide concentrations by freeze-drying the leaf used to assess latex exudation and its opposite at − 80 °C, then finely grinding 50 mg of pooled tissue per population and following the protocol described in Petschenka et al.^77^.

In mid-July and mid-August 2020, we surveyed the abundance of ten specialist herbivore species located on all aboveground parts of the plants (Fig. 2). The total number of leaf splotch mines represented the abundance of the milkweed leaf-mining fly. Eggs, caterpillars, chrysalises, and butterflies (i.e., all life stages) contributed towards Monarch butterfly abundance. In 2021, we repeated the surveys of three species: the Monarch butterfly, the milkweed leaf-mining fly, and the swamp milkweed leaf beetle (*Labidomera clivicollis*).

We assessed eight traits associated with plant growth and development. We measured the height of all ramets before and after flowering began as the length of the ramet from soil level to the apical meristem, a surrogate of ramet biomass^49^. Plant mortality was recorded in early September, and we counted the number of ramets per pot before and after flowering began as each single shoot emerging from the soil or a shoot connected to another shoot at the soil surface. We calculated relative growth rate as:

$${\text{RGR}} = \frac{{{\text{log}}\left({{\text{height}}\;{\text{after}}\;{\text{flowering}}} \right) - \text{log} \left( {{\text{height}}\;{\text{before}}\;{\text{flowering}}} \right)}}{{{\text{Days}}\;{\text{between}}\;{\text{measurements}}}}$$

To assess specific leaf area (SLA) and leaf dry matter content (LDMC), we collected the youngest fully expanded intact leaf and measured wet mass, dry mass, length, and width. SLA and LDMC were calculated as leaf area/dry mass (m^2^/g) and dry mass/wet mass, respectively.

We assessed nine traits associated with plant reproduction. Flowering success represented whether a plant produced any fully reflexed flowers. We recorded flowering duration as the number of days between the dates of the first fully reflexed flowers on the oldest and youngest inflorescences (i.e., flower clusters). For the three oldest inflorescences per plant, we recorded the number of flowers per inflorescence once at least half the flowers in an inflorescence became fully reflexed. From three flowers per inflorescence, we assessed the number of pollinaria removed and flower size. Flower size was measured as the length and width of each flower’s corolla (petal), hood (nectar-containing structure), and distance from one hood tip to the opposite side of the reproductive whorl (Supplementary Fig. S3). We counted the total number of inflorescences produced throughout the entire growing season. We recorded the date of the first mature follicle per plant when it exceeded 5 cm in length as smaller follicles are the most likely to be aborted^56^, as well as the total number of mature follicles per plant.

### Statistical analyses

#### Quantitative genetic parameters

All analyses were performed using R v.4.1.1^78^.

We calculated three quantitative genetic parameters to understand how urbanization could affect divergence between populations and genetic variation within populations. To estimate the variances explained by population and family for each trait, we used the *glmmTMB* package^79^ (version 1.1.2.2) to fit the following general linear models using restricted maximum likelihood:

$${\text{Response}}\;{\text{variable}}\sim {\text{Block}} + \left( {1|{\text{Population}}/{\text{Family}}} \right)$$

In these models, data was restricted to the last year of measurement to minimize impacts of maternal effects. Block was treated as a fixed effect while Population and Family were random effects, with Family nested within Population.

We extracted population and family-level variances with the “VarCorr” function from the *lme4* package^80^ (version 1.1.27.1), and residual variances with the “sigma” function from the *stats* package^78^ (version 4.1.1).

We calculated estimates of full-sibling broad-sense heritability (*H*^2^), the ratio of genetic variance to total phenotypic variance^50^, for each phenotypic trait as:

$$H^{2} = \frac{{{\text{genetic}}\;{\text{variance}}}}{{{\text{total}}\;{\text{phenotypic}}\;{\text{variance}}}}$$

where genetic variance for full-sibs was calculated as (family-level variance)$\times$ 2 and total phenotypic variance was calculated as family-level variance + population-level variance + residual variance. We calculated Q_ST_, a standardized measure of the genetic differentiation of a quantitative trait among populations^81^, as:

$${\text{Q}}_{{{\text{ST}}}} = \frac{{{\text{population}} - {\text{level}}\;{\text{variance}}}}{{{\text{population}} - {\text{level}}\;{\text{variance}} + {\text{genetic}}\;{\text{variance}} \times 2{ }}}$$

We calculated the coefficient of genetic variation (CV_G_), a dimensionless measure of evolutionary potential that is closely related to the coefficient of additive genetic variation (CV_A_) and is useful for comparing the magnitude of genetic variation among traits^82^, as:

$${\text{CV}}_{{\text{G}}} = \frac{{{\text{ genetic}}\;{\text{variance}}^{0.5} }}{{{\text{trait}}\;{\text{mean}}}}$$

#### Genetic variation within and between populations (Q1)

To quantify the statistical significance of heritable genetic variation for phenotypic traits within and between populations, we refitted the models described above with the “lmer” and “glmer” functions from the *lme4* package, for response variables with Gaussian and non-Gaussian distributions, respectively. This step was necessary because the original models fit with *glmmTMB* were not compatible with the “ranova” and “PBmodcomp” functions described below.

For models with Gaussian distributions, we tested the significance of Population and Family using the “ranova” function from the *lmerTest* package^83^ (version 3.1.3), then divided *p*-values by 2 because these were 1-sided tests (i.e., variance ≥ 0)^84^. We then obtained percent variance explained (PVE) for each random effect after extracting variances with *lme4* and calculating:

$${\text{PVE}} = \frac{{{\text{random}}\;{\text{effect}}\;{\text{variance}}}}{{{\text{random}}\;{\text{effect}}\;{\text{variance}} + {\text{residual}}\;{\text{variance}}}}$$

For models with non-Gaussian distributions, we tested the significance of Population and Family using the “PBmodcomp” function from the *pbkrtest* package^85^ (version 0.5.1) with 1000 simulations, then divided *p*-values, which were calculated assuming that the likelihood ratio test has a χ^2^ distribution, by 2 because these were 1-sided tests (i.e., variance ≥ 0)^84^. Results for ten models analyzed with 1000 simulations were identical to those analyzed with 100, so we used 100 simulations for the remaining models. We refitted models to Gaussian distributions, then extracted variances and calculated PVE for each random effect as described above. We inspected model diagnostics with the *DHARMa*^86^ (version 0.4.3) and *performance*^87^ (version 0.7.3) packages and transformed response variables to meet the assumptions of normality and homogeneity of variance when necessary (Supplementary Tables S1-2). Response variables associated with plant herbivory and milkweed stem weevil damage were analyzed with hurdle models^88^ manually to account for an excess of zeroes as a combination of two separate models: one to evaluate if herbivory/damage was present and the other to quantify the damage.

To test whether the number of phenotypic traits with heritable genetic variation within and between populations was significantly different from the expected number of these traits, we performed binomial expansion tests^89,90^ with the “binom.test” function from the *stats*^78^ package (version 4.1.1). Because we set alpha to 0.05 for the previous hypothesis tests of individual traits, we set the probability of success to 0.05. The tests were one-sided (alternative = “greater”) to identify scenarios wherein the proportion of “successes” (traits with heritable genetic variation) out of all “trials” (all traits) was significantly higher than the expected proportion. Since this test functions under the assumption of independent trials, and there are inherent correlations among traits (Supplementary Figures S4-S5), we acknowledge that these correlations could inflate type I error and should be interpreted with this caveat.

#### Genetic clines along an urban–rural gradient (Q2)

To test if urbanization caused genetic divergence among populations, we fitted linear mixed effect models with two metrics of urbanization added as fixed effects using the same packages described above. For both general and generalized linear mixed effect models, urbanization was added to models as both Distance to the City Center and Urbanization Score, separately. To test the significance of random effects (i.e., Population & Family), we repeated the remaining steps as described for Question 1 above. If urbanization contributed to heritable genetic variation within and/or between populations (i.e., *p*-values for Question 1 models were significant for Family and/or Population), then these *p*-values should have been higher in Question 2 models’ results. To test the significance of fixed effects (i.e., urbanization), we adjusted the models to use maximum likelihood and then computed χ^2^ and *p*-values from a type II sums-of-squares ANOVA with the *car* package^91^ (version 3.0.11). We used type II sums-of-squares because this method is unbiased by the order of effects, especially for unbalanced data, unlike type I. In contrast to type III, type II has more statistical power and is based on the assumption that interactions are minimal or absent^92^. If urbanization contributed to genetic variation within and/or between populations, then some variance should have shifted from Population and/or Family to urbanization and the *p*-values for urbanization should have become significant.

We performed this analysis for each trait, regardless of whether it showed a significant effect of population, to complement our initial test for population divergence. For the first test, we evaluated whether the variance among populations, treated as a categorical factor, was greater than zero. However, urbanization can also be treated as a quantitative metric in a regression framework to test for clines in traits. This second analysis allowed us to potentially tease out more subtle quantitative differences among populations that are harder to detect when populations are treated as categorical factors. Thus, the latter analysis is an important complement to the first even when there is no initial evidence of population differentiation.

To test for differences associated with urbanization in overall phenotype, as opposed to differences in individual traits that could reflect specific selection pressures, we performed a multivariate phenotype analysis. This analysis incorporated all traits in the dataset and accounted for non-independence between correlated traits (Supplementary Figures S4-S5). We computed best linear unbiased predictions (BLUPs) for each model with the “ranef” function from *lme4*, placed these values in a response matrix, and then used the *mvabund* package^93^ (version 4.2.1) to fit general linear models and examine how multivariate phenotype varied with urbanization (both Distance and Urbanization Score) using one-way ANOVA.

#### Genetic differentiation between a green corridor and urban matrix (Q3)

To evaluate how genetic variation was associated with a green corridor between urban populations (“Urban: Corridor” and “Urban: Non-Corridor”), we added Subtransect and Urbanization:Subtransect interaction terms to the linear mixed effect models described for Question 2. For both general and generalized linear mixed effect models, we tested the significance of the random effects (i.e., Population & Family) by repeating the steps as described for Question 2 above. To test the significance of the fixed effects (i.e., Urbanization, Subtransect, and their interaction), we fitted reduced models without the interaction term, ranked full and reduced models based on Akaike's information criterion (AIC)^94^, and selected the model with the lowest AIC as the best model. We adjusted the models to use maximum likelihood and then computed χ^2^ and *p*-values from a type III sums-of-squares ANOVA if the best model contained an interaction with *p* ≤ 0.1 because this method tests for main effects after testing for interactions^92^; otherwise, we reran the analyses with type II sums-of-squares.

We also fitted models with multiple years of data and found that the effects of urbanization and a green corridor were qualitatively identical to those reported in Tables 2, 3 in 85% of cases (Supplementary Text, Supplementary Table S3). We then repeated the multivariate analyses for these models as described for Question 2. Cardenolides were not analyzed due to there being a low sample size among urban populations.

**Table 2:** Results from general and generalized linear mixed models examining the effects of urbanization on all phenotypic traits.

	Distance		Urbanization score		Individuals	Populations
	χ^2^	*p*	χ^2^	*p*		
**Defense/damage**						
Latex exudation	0.245	0.620	4.029	**0.045**	699	51
Herbivory before flowering (binary)	2.964	0.085	0.438	0.508	794	51
Herbivory before flowering (quantitative)	3.605	0.058	6.221	**0.013**	430	50
Herbivory after flowering (binary)	0.851	0.356	0.614	0.433	686	51
Herbivory after flowering (quantitative)	1.045	0.307	0.020	0.887	661	51
Milkweed stem weevil damage (binary)	0.715	0.398	0.045	0.832	938	52
Milkweed stem weevil damage (quantitative)	1.611	0.204	0.579	0.447	685	51
**Reproduction**						
Flowering success	0.001	0.978	0.024	0.876	938	52
Flowers per inflorescence	2.104	0.147	3.552	0.059	178	47
Flower size	0.602	0.438	0.794	0.373	175	46
Flowering duration	0.006	0.937	0.034	0.853	178	47
Date of first flower	0.117	0.733	0.416	0.519	178	47
Follicles	0.014	0.906	0.088	0.767	178	47
Pollinaria removed	3.592	0.058	3.289	0.070	175	46
Date of first follicle	0.369	0.543	0.001	0.981	126	42
Inflorescences	0.016	0.899	0.254	0.614	177	47
**Herbivore abundance**						
Monarch butterfly abundance	1.545	0.214	1.987	0.159	939	52
Milkweed leaf-mining fly abundance	0.092	0.762	0.666	0.414	939	52
Swamp milkweed leaf beetle abundance	0.017	0.898	0.198	0.656	939	52
**Growth**						
LDMC	1.959	0.162	0.261	0.609	795	51
SLA	0.100	0.752	0.135	0.714	772	51
Height before flowering	1.275	0.259	0.304	0.581	822	51
Height after flowering	1.328	0.249	0.358	0.550	939	52
Relative growth rate	0.000	0.990	0.151	0.697	568	51
Ramets before flowering	1.886	0.170	0.080	0.777	939	52
Ramets after flowering	1.876	0.171	0.052	0.820	939	52
Mortality	0.104	0.747	0.204	0.652	938	52

All populations were included. Shown are maximum likelihood χ^2^ and *p*-values obtained from type II sums-of-squares ANOVA. Though not shown, block was included as a fixed effect and often explained significant variation in the common garden. Significant *p*-values (*p* ≤ 0.05) are bolded. Relative growth rate was only analyzed for plants exhibiting positive growth between measurements.

**Table 3:** Results from general and generalized linear mixed models examining the effects of urbanization and proximity to the urban green corridor on all phenotypic traits.

	Distance		Subtransect		D × S		Individuals	Populations
	χ^2^	*p*	χ^2^	*p*	χ^2^	*p*		
**Defense/damage**								
Latex exudation	1.612	0.204	0.014	0.906			474	35
Herbivory before flowering (binary)	1.023	0.312	0.462	0.497			534	35
Herbivory before flowering (quantitative)	0.054	0.817	0.122	0.727			303	35
Herbivory after flowering (binary)	0.581	0.446	0.672	0.412			466	35
Herbivory after flowering (quantitative)	0.004	0.949	0.389	0.533			450	35
Milkweed stem weevil damage (binary)	3.572	0.059	0.166	0.684			635	36
Milkweed stem weevil damage (quantitative)	0.082	0.774	0.829	0.363			464	35
**Reproduction**								
Flowering success	0.095	0.757	0.625	0.429			634	36
Flowers per inflorescence	0.546	0.460	4.246	**0.039**			126	33
Flower size	1.374	0.241	0.576	0.448			124	33
Flowering duration	0.410	0.522	2.027	0.155			126	33
Date of first flower	0.725	0.394	0.006	0.939			126	33
Pollinaria removed	0.379	0.538	0.012	0.912			124	33
Follicles	0.003	0.953	0.580	0.446	0.098	0.755	126	33
Date of first follicle	3.306	0.069	0.944	0.331			90	30
Inflorescences	0.199	0.656	0.454	0.501			125	33
**Herbivore abundance**								
Monarch butterfly abundance	4.697	**0.030**	0.158	0.691			635	36
Milkweed leaf-mining fly abundance	0.290	0.590	0.168	0.681			635	36
Swamp milkweed leaf beetle abundance	0.404	0.525	0.068	0.794			635	36
**Growth**								
LDMC	2.710	0.100	1.456	0.228			533	35
SLA	0.400	0.527	0.840	0.360			516	35
Height before flowering	0.126	0.722	0.009	0.925			555	35
Height after flowering	3.484	0.062	0.056	0.813	2.366	0.124	635	36
Relative growth rate	0.036	0.849	0.564	0.453			392	35
Ramets before flowering	2.861	0.091	0.002	0.962	2.050	0.152	635	36
Ramets after flowering	6.005	**0.014**	3.231	0.072	2.740	0.098	635	36
Mortality	1.089	0.297	0.062	0.803			634	36

Urbanization was quantified via distance from the urban center and only urban populations were included. Shown are maximum likelihood χ^2^ and *p*-values obtained from type II and III sums-of-squares ANOVA. Though not shown, block was included as a fixed effect and often explained significant variation in the common garden. Significant *p*-values (*p* ≤ 0.05) are bolded. Relative growth rate was only analyzed for plants exhibiting positive growth between measurements.

We did not perform phenotypic or genotypic selection analyses because our experiment was only designed to quantify quantitative genetic variation. We could not obtain accurate estimates of variation in lifetime fitness because common milkweed is a long-lived perennial (members of this genus can live for over two decades^51^). Moreover, since our common garden was located in a rural area, we could only measure selection in one environment. It would have been more appropriate to measure selection if we had comparisons among urban and rural environments, but this would have required planting multiple common gardens.

## Results

### Genetic variation within and between populations (Q1)

There was heritable variation for multiple phenotypic traits within populations and evidence of genetic differentiation between populations for some traits. At least one trait per category exhibited significant heritable genetic variation within populations (nine traits, total). Heritabilities and coefficients of genetic variation ranged from 0–0.588 (mean ± SE: 0.110 ± 0.031) and 0–4.456 (mean ± SE: 0.467 ± 0.207), respectively (Table 1, Supplementary Table S4). The highest statistically significant heritabilities were observed for herbivory after flowering (binary) (*H*^2^ = 0.588, *p* = 0.004), pollinaria removed (*H*^2^ = 0.544, *p* = 0.034), and milkweed stem weevil damage (binary) (*H*^2^ = 0.418, *p* = 0.030), while nine traits, at least one per category, exhibited near-zero heritabilities. Milkweed stem weevil damage (binary) (CV_G_ = 0.995, *p* = 0.030), herbivory after flowering (binary) (CV_G_ = 0.948, *p* = 0.004), and Monarch butterfly abundance (CV_G_ = 0.767, *p* = 0.021) exhibited the highest, statistically significant coefficients of genetic variation, while seven traits exhibited near-zero values. It was unlikely that the high frequency of heritable genetic variation within populations was due to chance (binomial expansion test: *p* < 0.001). Three traits associated with plant defense and reproduction exhibited statistically significant genetic divergence among populations: latex exudation (Q_ST_ = 0.174, *p* = 0.016), flowering success (i.e., whether plants flowered) (Q_ST_ = 0.492, *p* = 0.014), and no. of inflorescences (Q_ST_ = 1, *p* = 0.035) (Table 1, Supplementary Table S4). Q_ST_ ranged from 0 to 1 (mean ± SE: 0.241 ± 0.068) with seven traits exhibiting near-zero values. The relatively few instances of genetic divergence between populations could be due to chance (binomial expansion test: *p* = 0.150). Thus, these results suggest moderate heritable genetic variation within populations and little phenotypic divergence among populations, the first of which is a prerequisite for adaptation.

### Genetic clines along an urban–rural gradient (Q2)

**Figure 3:**
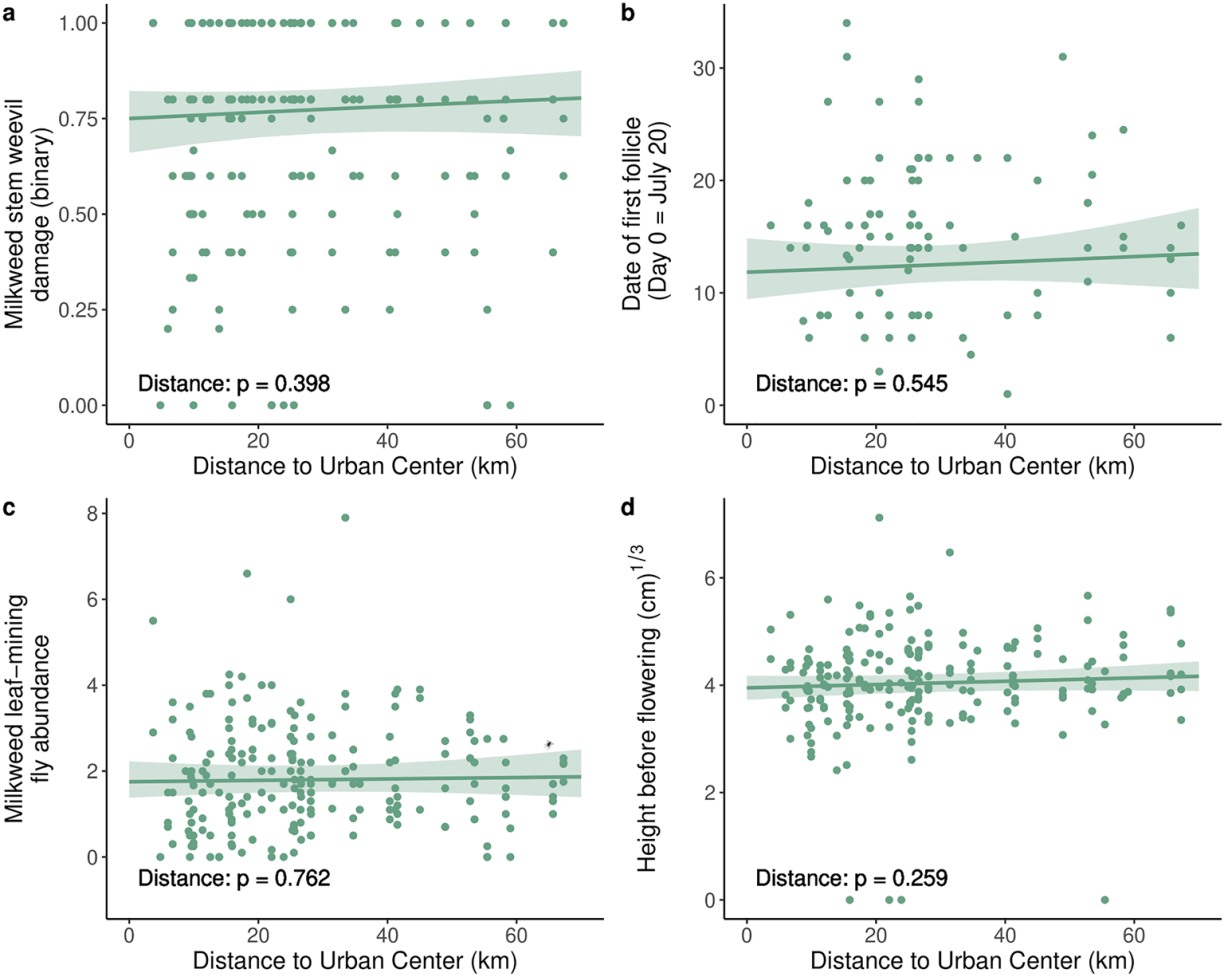
The effect of urbanization on representative traits from each main category: (**a**) plant defense/damage (e.g., the presence of milkweed stem weevil damage); (**b**) plant reproduction (e.g., date of first follicle); (**c**) herbivore abundance (e.g., milkweed leaf-mining fly abundance); and (**d**) plant growth (e.g., height before flowering) when urbanization was quantified by distance from the urban center and all populations were included. Regression lines represent predicted values with a 95% confidence interval and points which represent family-level means are shown for general and generalized linear mixed effects models. These traits were modelled using the following distributions: binomial (**a**), negative binomial (**b**, **c**), and Gaussian (**d**).

We found little evidence for genetic clines along the urbanization gradient studied. When quantified as distance to the city center, urbanization did not significantly impact phenotypic traits (Fig. 3, Supplementary Figures S6-S14, Table 2, Supplementary Tables S6–S8). When quantified as an urbanization score, we detected relationships with latex exudation (χ^2^ = 4.029, *p* = 0.045, R^2^_m_ = 0.037, N = 699 individuals & 51 populations) and herbivory before flowering (quantitative) (χ^2^ = 6.221, *p* = 0.013, R^2^_m_ = 0.010, N = 430 individuals & 50 populations). When adjusted for false discovery rates (i.e., controlling for type I error with the Benjamini–Hochberg procedure), our results did not provide strong evidence that urbanization influenced genetic divergence in phenotypic traits between populations (Supplementary Tables S9-10). Moreover, the multivariate analysis indicated that urbanization did not impact overall phenotype when urbanization was quantified as distance to the city center (F_1,40_ = 14.771, *p* = 0.711) or urbanization score (F_1,40_ = 6.006, *p* = 0.984). Consistent with our results that urbanization had little effect on genetic divergence, the variation explained and statistical significance of the effects of population and genetic family did not substantially change once urban metrics were included in models (Supplementary Table S5). Taken together, these multiple lines of testing reveal little support for genetic divergence along an urbanization gradient in phenotypic traits in common milkweed.

### Genetic differentiation between a green corridor and urban matrix (Q3)

**Figure 4:**
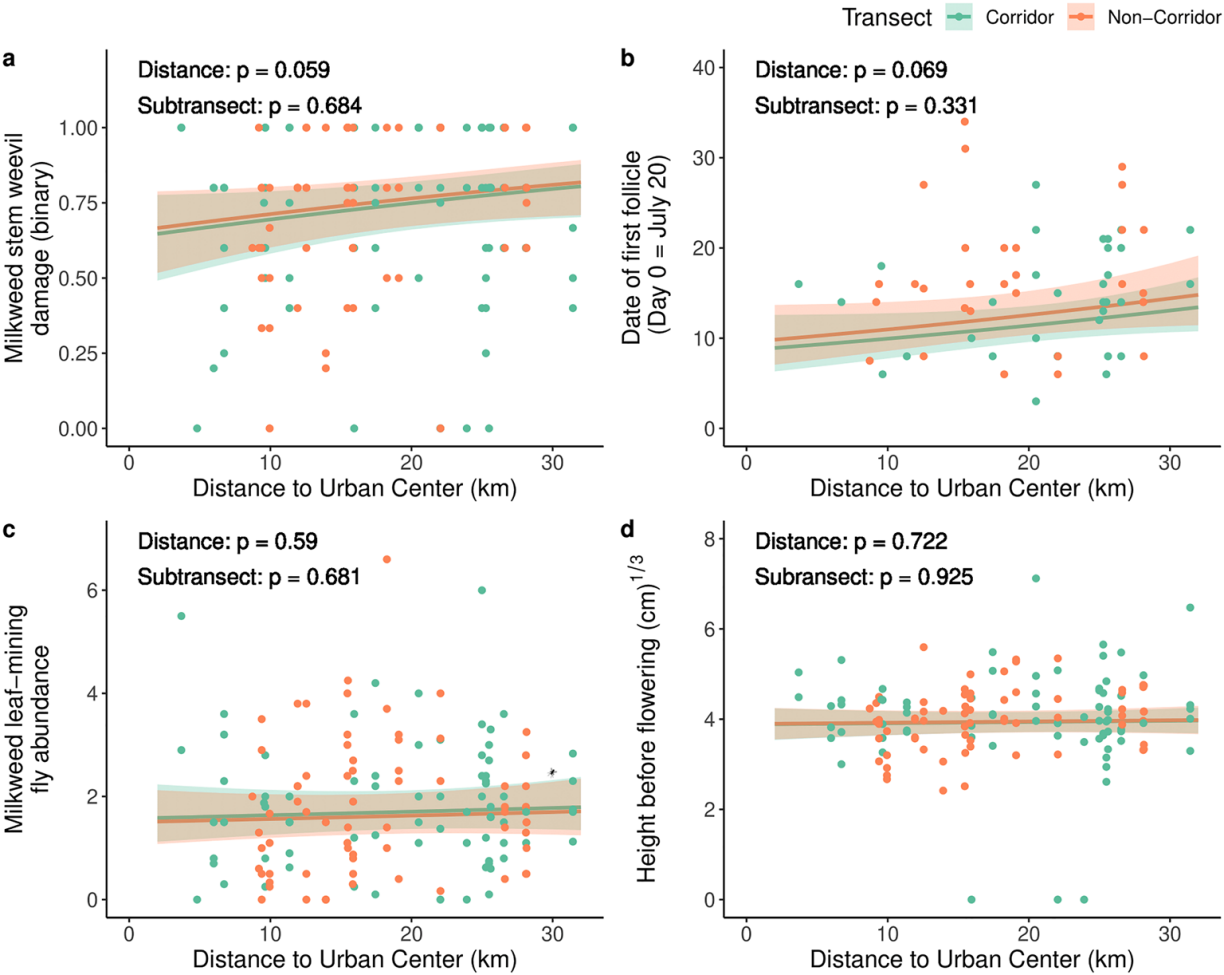
The effects of urbanization and proximity to a green corridor on representative traits from each main category: (**a**) plant defense/damage (e.g., the presence of milkweed stem weevil damage); (**b**) plant reproduction (e.g., date of first follicle); (**c**) herbivore abundance (e.g., milkweed leaf-mining fly abundance); and (**d**) plant growth (e.g., height before flowering) when urbanization was quantified by distance from the urban center and only urban populations were included. Regression lines represent predicted values with a 95% confidence interval and points which represent family-level means are shown for general and generalized linear mixed effects models. These traits were modelled using the following distributions: binomial (**a**), negative binomial (**b**, **c**), and Gaussian (**d**).

Proximity to a green corridor did not strongly influence genetic divergence in phenotypic traits among populations, or the presence of clines along an urbanization gradient (Fig. 4, Supplementary Figures S15-S23, Table 3, Supplementary Tables S7 & S12). In the single-trait analysis, proximity to a green corridor had a statistically significant yet very small impact on flowers per inflorescence when urbanization was quantified as distance to the city center (χ^2^ = 4.246, *p* = 0.039, R^2^_m_ < 0.001, N = 126 individuals & 33 populations) and urbanization score (χ^2^ = 5.572, *p* = 0.018, R^2^_m_ < 0.001, N = 126 individuals & 33 populations). We detected an interaction between urbanization (quantified as an urbanization score) and proximity to a green corridor for latex exudation (χ^2^ = 5.164, *p* = 0.023, R^2^_m_ = 0.060, N = 474 individuals & 35 populations), flowering duration (χ^2^ = 7.614, *p* = 0.006, R^2^_m_ = 0.003, N = 126 individuals & 33 populations), follicles (χ^2^ = 7.454, *p* = 0.006, R^2^_m_ = 0.325, N = 126 individuals & 33 populations), date of first follicle (χ^2^ = 4.467, *p* = 0.035, R^2^_m_ = 0.167, N = 90 individuals & 30 populations), inflorescences (χ^2^ = 5.252, *p* = 0.022, R^2^_m_ < 0.001, N = 125 individuals & 33 populations), and milkweed stem weevil damage (binary) (χ^2^ = 4.667, *p* = 0.031, R^2^_m_ = 0.034, N = 635 individuals & 36 populations). These interactions suggest that the impact of corridors may depend on the intensity of urbanization. However, when adjusted for false discovery rates, our results did not provide strong evidence that proximity to a green corridor influenced genetic divergence in phenotypic traits between populations as none of these effects were statistically significant (Supplementary Tables S13-14). Additionally, the multivariate analysis indicated that proximity to a green corridor did not impact overall phenotypic divergence when urbanization was quantified as distance to the city center (F_1,27_ = 0.346, *p* = 1.000) or urbanization score (F_1,27_ = 0.756, *p* = 1.000). Consistent with our results that proximity to a green corridor had little effect on genetic divergence, the variation explained and significance of the effects of population and genetic family did not substantially change once urban metrics and proximity to a green corridor were included in models (Supplementary Table S11). These results also reveal little support that proximity to a green corridor is associated with genetic divergence among urban common milkweed populations.

## Discussion

In this study, we tested the hypotheses that urbanization and a single urban green corridor drive genetic divergence in phenotypic traits among populations of common milkweed. Though we observed moderate heritable genetic variation within populations and some weak phenotypic divergence among populations, we found low support that urbanization or proximity to an urban green corridor influenced genetic divergence. These results suggest that common milkweed has not undergone rapid evolutionary change in response to urban landscapes as a whole, nor a common component of urban environments: a green corridor. Despite these results, we maintain that our study system and experimental design are well-suited for addressing our research questions and that these findings are important for developing an accurate understanding of how urbanization impacts evolution. Below, we discuss the implications of these results for understanding how species evolve in response to rapid environmental change in heterogeneous urban environments.

### Genetic variation within and between populations (Q1)

Genetic divergence of phenotypic traits along an urbanization gradient necessitates that populations exhibit genetic divergence, which is more likely to occur when those populations contain heritable phenotypic variation. These criteria have been observed in previous studies of common milkweed, although not in an urban context^64–68,95^. For instance, many phenotypic traits are heritable, especially those associated with defense/damage and growth; examples include *Tetraopes* spp. damage, carbon:nitrogen ratio, SLA, water percentage, trichome density, ant abundance, root and shoot mass, constitutive and induced latex and cardenolides^64–68,95^. In addition, substantial heritable phenotypic variation within populations was found at both a continental scale (i.e., within populations sampled across the species’ native North American range^96^) and a local scale (i.e., within single genetic populations^65,66^, but see Potts and Hunter^97^). Similarly, genetic divergence for growth-related traits was detected among common milkweed populations sampled from a > 1500 km latitudinal gradient from New Brunswick, Canada, to North Carolina, USA^68^. In our common garden experiment, we found moderate heritable genetic variation within populations and weak phenotypic divergence among populations sampled from an urbanization gradient. Our results, which indicate that these populations have met the prerequisites to evolve in response to urbanization and/or an urban green corridor, are mostly consistent with previous findings from non-urban contexts^64,67^.

Our estimates of genetic variation for phenotypic traits within populations are comparable to previous studies^64,67^. For example, heritability estimates for plant height after flowering and the number of ramets (range: *H*^2^ = 0.132–0.161) were close to estimates by Vannette et al.^67^ (aboveground biomass: *H*^2^ = 0.11) and Agrawal et al.^64^ (vegetative biomass: *H*^2^ = 0.12). We found significant and relatively high heritability estimates for herbivory after flowering (*H*^2^ = 0.588) and milkweed stem weevil damage (*H*^2^ = 0.418) when measured on a presence/absence basis, but not quantitatively when measured as percent leaf area removed. In comparison, Agrawal et al.^64^ found moderate heritability for herbivory when measured as the percentage of leaves with foliar damage due to chewing herbivores (*H*^2^ = 0.284), but not for milkweed stem weevil damage when measured as the length of stem scars (*H*^2^ = 0.037). We also found that two reproductive traits had moderate to high heritabilities (i.e., pollinaria removed: *H*^2^ = 0.544; date of first follicle: *H*^2^ = 0.235), suggesting a higher evolvability of these traits in response to environmental change. Overall, these results confirm the capacity for these common milkweed populations to evolve in response to ecological disturbances, such as urbanization.

### Genetic clines along an urban–rural gradient (Q2)

Many taxa exhibit genetic divergence between urban and nonurban populations for various traits. In plants, this has been documented for traits associated with phenology^16,27,29,30^, size^16,29^, fecundity^16,29^, defense^16^, and competitive ability^98^. Additionally, in both native and introduced ranges, common milkweed exhibits clines for growth and leaf physiology traits that correspond with a defining feature of urban environments: temperature^96^. Yet despite surveying several suites of traits in common milkweed, we found low support for genetic divergence along an urbanization gradient. Furthermore, we detected only small effect sizes (range: 0.01–0.037) for the few traits associated with urbanization even with the large scale and replication afforded by our experimental design. Thus, multiple lines of evidence suggest the lack of such divergence at present, though we do not, and cannot, rule out the possibility of genetic divergence emerging in the future.

The relative rarity of this outcome in the existing urban evolution literature presents a valuable opportunity to explore circumstances that could prevent urbanization from influencing genetic divergence among populations. In this case, evolutionary change may have been precluded by urban ecological pressures that were possibly too small or brief, as the city of Toronto has contained ≥ 50,000 residents for only ca. 150 years^99^. The life history traits of common milkweed could also slow evolutionary change. For example, the vegetative reproduction inherent to common milkweed can lead to clonal growth and the loss of genotypic diversity within populations^100,101^, which is compounded by the species’ long-lived nature. Long-distance pollen and seed flow could yield high gene flow among populations and low genetic drift within populations, as common milkweed is largely self-incompatible (i.e., self-pollinated plants only produce viable fruit at very low rates^102^) , seeds are wind-dispersed, and flowers are frequently pollinated by insects that can travel > 1 km (e.g., *Bombus* spp*., Apis mellifera*)^103,104^.

We acknowledge that urban-nonurban environmental gradients are complex and that our data cannot fully capture the heterogeneity of the landscape mosaic. Urbanization is a multifaceted process that involves complex change along axes including, but not limited to, environmental, economic, and sociological dimensions. There are manifold ways to define “urban” vs. “rural” vs. “natural” landscapes^105^, and environmental heterogeneity functions across temporal and spatial scales^106,107^. However, our methods account for both temporal and spatial processes. Distance from the city center is highly correlated with percent impervious surface and numerous other environmental variables associated with urbanization^69^, and in Toronto reflects urban expansion in concentric zones, with each zone’s environment correlated with the time since development. By including urban and rural environments as transect endpoints and densely-sampled transitional areas in between, distance from the urban center captures extensive multivariate environmental change associated with urbanization— an important feature, since we did not know which environmental factors might impact common milkweed a priori. A complementary metric is urbanization score, which quantifies the immediate land use/land cover surrounding each population, without consideration of the site’s proximity to the urban core. Therefore, despite this limitation, we argue that our estimates of urbanization are credible and reliable.

This study lends valuable context to prior work demonstrating how urbanization impacts the reproductive success of common milkweed populations^49^. Specifically, as we observed relatively little phenotypic divergence among populations within the common garden, our results suggest that the previously observed *in-situ* phenotypic divergence was mostly consistent with phenotypic plasticity as opposed to genetic divergence. This finding underscores the importance of investigating the genetic basis of phenotypic divergence observed in cities and reiterates the role of phenotypic plasticity in shaping how populations respond to human-caused change. Future studies could investigate the specific conditions that promote phenotypic plasticity within urban environments.

### Genetic differentiation between a green corridor and urban matrix (Q3)

Very little is known about how urban green corridors influence genetic divergence among plant populations. In non-urban environments, green corridors are predicted to increase gene flow among plant populations by facilitating pollen flow and seed dispersal, which is expected to decrease genetic divergence between populations^108–110^. Limited research in urban environments suggests that green corridors often increase gene flow among animal populations^46,111–113^ (but see Angold et al.^114^), and that urban features such as railways can function as corridors among plant and animal populations^115,116^. In our study, proximity to a green corridor did not strongly influence genetic divergence in phenotypic traits among common milkweed populations. Despite finding low support for genetic divergence along an urbanization gradient in Question 2, we proceeded with this analysis because we believe that once research questions or hypotheses have been decided, it is important to address each, so as not to change the hypothesis based on the results.

As discussed above for Question 2, urbanization may not restrict gene flow among populations. If true, there would be little opportunity for the green corridor to significantly enhance gene flow and diminish any hypothetical genetic divergence in common milkweed, as proposed for butterflies in Angold et al.^114^. Conversely, our data also suggest that proximity to a green corridor does not inherently facilitate genetic divergence either. It is also possible that urban green corridors actually impact genetic divergence but that our results reflect our inclusion of a single corridor, or the specific environmental conditions associated with our chosen corridor. For example, the efficacy of the corridor could have been impeded by a highly hostile external matrix or edge effects generated by the narrow shape^117,118^. Relatedly, the corridor we sampled would have provided minimal connectivity to plants or pollen vectors if it were not perceived as a functional corridor to either group–the plants or the pollinators. The manner in which common milkweed established in this particular corridor also influences our results, as plants growing in the corridor since establishment many generations ago are more likely to exhibit genetic divergence than plants descending from relatives in nearby non-corridor environments. Regardless, these findings contradict the expectation that habitat fragmentation impacts the evolutionary processes of urban populations and invite further research into how and when corridors impact genetic divergence of phenotypic traits in urban populations.

### Conclusion

Urbanization is associated with phenotypic trait divergence for many species, yet in most cases, the genetic basis of these trait differences is unresolved. Additionally, the extent to which specific aspects of the urban landscape impact genetic divergence in phenotypic traits among plant populations is virtually unknown. Here, we show that neither urbanization nor an urban green corridor impacted genetic divergence in phenotypic traits in common milkweed, a native plant of conservation importance. These results demonstrate an example in which our measures of urbanization have not substantially contributed to genetic divergence among populations. To our knowledge, this study is also the first to investigate if urban green corridors impact genetic divergence in phenotypic traits in plant populations and ultimately suggests the absence of such effects in our study system. To further understand how urban environments and urban green corridors impact eco-evolutionary dynamics in plant populations, future research should verify the consistency of these findings by testing similar questions in plants with diverse life history, and in a range of cities with heterogeneous landscapes.

### Supplementary Information

Supplementary Information 1.

Supplementary Information 2.

## Data Availability

Data are archived on Zenodo (https://doi.org/10.5281/zenodo.7615892). Code is available on Zenodo (https://zenodo.org/doi/10.5281/zenodo.10145007) and on the GitHub page for S.T.B. (https://github.com/sbreitbart/urban_genetic_divergence).

## References

[1] Grimm NB (2008). Global change and the ecology of cities. Science.

[2] McDonnell MJ, MacGregor-Fors I (2016). The ecological future of cities. Science.

[3] McKinney ML (2008). Effects of urbanization on species richness: A review of plants and animals. Urban Ecosyst..

[4] Concepción ED (2016). Impacts of urban sprawl on species richness of plants, butterflies, gastropods and birds: Not only built-up area matters. Urban Ecosyst..

[5] Saari S (2016). Urbanization is not associated with increased abundance or decreased richness of terrestrial animals—Dissecting the literature through meta-analysis. Urban Ecosyst..

[6] Piano E (2020). Urbanization drives cross-taxon declines in abundance and diversity at multiple spatial scales. Glob. Chang. Biol..

[7] Ordeñana MA (2010). Effects of urbanization on carnivore species distribution and richness. J. Mammal..

[8] Gaynor KM, Hojnowski CE, Carter NH, Brashares JS (2018). The influence of human disturbance on wildlife nocturnality. Science.

[9] Miles LS, Breitbart ST, Wagner HH, Johnson MTJ (2019). Urbanization shapes the ecology and evolution of plant-arthropod herbivore interactions. Front. Ecol. Evol.

[10] Murray-Stoker D, Johnson MTJ (2021). Ecological consequences of urbanization on a legume–rhizobia mutualism. Oikos.

[11] Theodorou P (2022). The effects of urbanisation on ecological interactions. Curr. Opin. Insect Sci..

[12] Barrett K, Helms BS, Samoray ST, Guyer C (2010). Growth patterns of a stream vertebrate differ between urban and forested catchments. Freshw. Biol..

[13] Evans KL (2012). Colonisation of urban environments is associated with reduced migratory behaviour, facilitating divergence from ancestral populations. Oikos.

[14] Alberti M, Marzluff J, Hunt VM (2017). Urban driven phenotypic changes: Empirical observations and theoretical implications for eco-evolutionary feedback. Philos. Trans. R. Soc. Lond. B Biol. Sci..

[15] Eggenberger H (2019). Urban bumblebees are smaller and more phenotypically diverse than their rural counterparts. J. Anim. Ecol..

[16] Santangelo JS, Rivkin LR, Advenard C, Thompson KA (2020). Multivariate phenotypic divergence along an urbanization gradient. Biol. Lett..

[17] Conner JK, Hartl DL (2004). A Primer of Ecological Genetics.

[18] Brans KI (2017). The heat is on: Genetic adaptation to urbanization mediated by thermal tolerance and body size. Glob. Chang. Biol..

[19] Johnson MTJ, Munshi-South J (2017). Evolution of life in urban environments. Science.

[20] Miles LS, Rivkin LR, Johnson MTJ, Munshi-South J, Verrelli BC (2019). Gene flow and genetic drift in urban environments. Mol. Ecol..

[21] Szulkin M, Munshi-South J, Charmantier A (2020). Urban Evolutionary Biology.

[22] Hitchings SP, Beebee TJ (1997). Genetic substructuring as a result of barriers to gene flow in urban *Rana temporaria* (common frog) populations: Implications for biodiversity conservation. Heredity.

[23] McClenaghan LR, Truesdale HD (2002). Genetic structure of endangered Stephens’ kangaroo rat populations in southern California. Southwest. Nat..

[24] Fusco NA, Pehek E, Munshi-South J (2021). Urbanization reduces gene flow but not genetic diversity of stream salamander populations in the New York City metropolitan area. Evol. Appl..

[25] Adducci A (2020). Urban coyotes are genetically distinct from coyotes in natural habitats. J. Urban Ecol..

[26] Cheptou P-O, Carrue O, Rouifed S, Cantarel A (2008). Rapid evolution of seed dispersal in an urban environment in the weed *Crepis sancta*. Proc. Natl. Acad. Sci. U. S. A..

[27] Lambrecht SC, Mahieu S, Cheptou PO (2016). Natural selection on plant physiological traits in an urban environment. Acta Oecol..

[28] Dubois J, Cheptou P-OO (2017). Effects of fragmentation on plant adaptation to urban environments. Philos. Trans. R. Soc. Lond. B Biol. Sci..

[29] Yakub M, Tiffin P (2017). Living in the city: Urban environments shape the evolution of a native annual plant. Glob. Chang. Biol..

[30] Gorton AJ, Moeller DA, Tiffin P (2018). Little plant, big city: A test of adaptation to urban environments in common ragweed (*Ambrosia artemisiifolia*). Proc. Biol. Sci..

[31] Johnson MTJ, Agrawal AA, Maron JL, Salminen J-P (2009). Heritability, covariation and natural selection on 24 traits of common evening primrose (*Oenothera biennis*) from a field experiment. J. Evol. Biol..

[32] Colautti RI, Eckert CG, Barrett SCH (2010). Evolutionary constraints on adaptive evolution during range expansion in an invasive plant. Proc. Biol. Sci..

[33] Gonzalez A, Lawton JH, Gilbert FS, Blackburn TM, Evans-Freke II (1998). Metapopulation dynamics, abundance, and distribution in a microecosystem. Science.

[34] Mech SG, Hallett JG (2001). Evaluating the effectiveness of corridors: A genetic approach. Conserv. Biol..

[35] Hale ML (2001). Impact of landscape management on the genetic structure of red squirrel populations. Science.

[36] Aars J, Ims RA (1999). The effect of habitat corridors on rates of transfer and interbreeding between vole demes. Ecology.

[37] Coulon A (2004). Landscape connectivity influences gene flow in a roe deer population inhabiting a fragmented landscape: An individual-based approach. Mol. Ecol..

[38] Christie MR, Knowles LL (2015). Habitat corridors facilitate genetic resilience irrespective of species dispersal abilities or population sizes. Evol. Appl..

[39] Orrock JL (2006). Conservation corridors affect the fixation of novel alleles. Conserv. Genet..

[40] Slatkin M (1987). Gene flow and the geographic structure of natural populations. Science.

[41] Barrett SCH, Charlesworth D (1991). Effects of a change in the level of inbreeding on the genetic load. Nature.

[42] Fowler K, Whitlock MC (1999). The variance in inbreeding depression and the recovery of fitness in bottlenecked populations. Proc. Biol. Sci..

[43] Hendry AP, Taylor EB, McPhail JD (2002). Adaptive divergence and the balance between selection and gene flow: Lake and stream stickleback in the Misty system. Evolution.

[44] Hirota T, Hirohata T, Mashima H, Satoh T, Obara Y (2004). Population structure of the large Japanese field mouse, *Apodemus speciosus* (Rodentia: Muridae), in suburban landscape, based on mitochondrial D-loop sequences. Mol. Ecol..

[45] Van Rossum F, Triest L (2012). Stepping-stone populations in linear landscape elements increase pollen dispersal between urban forest fragments. Plant Ecol. Evol..

[46] Munshi-South J (2012). Urban landscape genetics: Canopy cover predicts gene flow between white-footed mouse (*Peromyscus leucopus*) populations in New York City. Mol. Ecol..

[47] Lambert MR, Donihue CM (2020). Urban biodiversity management using evolutionary tools. Nat. Ecol. Evol.

[48] Miles LS, Murray-Stoker D, Nhan VJ, Johnson MTJ (2022). Effects of urbanization on specialist insect communities of milkweed are mediated by spatial and temporal variation. Ecosphere.

[49] Breitbart S, Tomchyshyn A, Wagner HH, Johnson MTJ (2023). Urbanization and a green corridor influence reproductive success and pollinators of common milkweed. Urban Ecosyst..

[50] Falconer DS, Mackay TFC (1996). Introduction to Quantitative Genetics.

[51] Wilbur HM (1976). Life history evolution in seven milkweeds of the genus *Asclepias*. J. Ecol..

[52] Bhowmik PC, Bandeen JD (1976). The biology of Canadian weeds: 19 *Asclepias syriaca* L.. Can. J. Plant Sci..

[53] Wyatt R, Broyles SB (1994). Ecology and evolution of reproduction in milkweeds. Annu. Rev. Ecol. Syst..

[54] MacIvor JS, Roberto AN, Sodhi DS, Onuferko TM, Cadotte MW (2017). Honey bees are the dominant diurnal pollinator of native milkweed in a large urban park. Ecol. Evol..

[55] Baker AM, Potter DA (2018). Colonization and usage of eight milkweed (*Asclepias*) species by monarch butterflies and bees in urban garden settings. J. Insect Conserv..

[56] Willson MF, Price PW (1977). The evolution of inflorescence size in *Asclepias* (Asclepiadaceae). Evolution.

[57] U.S. Fish and Wildlife Service. *Monarch (Danaus plexippus) Species Status Assessment Report V2.1*. (2020).

[58] Dussourd DE, Eisner T (1987). Vein-cutting behavior: Insect counterploy to the latex defense of plants. Science.

[59] Agrawal AA, Lajeunesse MJ, Fishbein M (2008). Evolution of latex and its constituent defensive chemistry in milkweeds (*Asclepias*): A phylogenetic test of plant defense escalation. Entomol. Exp. Appl..

[60] Agrawal AA, Hastings AP (2019). Plant defense by latex: Ecological genetics of inducibility in the milkweeds and a general review of mechanisms, evolution, and implications for agriculture. J. Chem. Ecol..

[61] Zhen Y, Aardema ML, Medina EM, Schumer M, Andolfatto P (2012). Parallel molecular evolution in an herbivore community. Science.

[62] Dobler S, Dalla S, Wagschal V, Agrawal AA (2012). Community-wide convergent evolution in insect adaptation to toxic cardenolides by substitutions in the Na, K-ATPase. Proc. Natl. Acad. Sci. U. S. A..

[63] Duffey SS (1980). Sequestration of plant natural products by insects. Annu. Rev. Entomol..

[64] Agrawal AA (2005). Natural selection on common milkweed (*Asclepias syriaca*) by a community of specialized insect herbivores. Evol. Ecol. Res..

[65] Mooney KA, Agrawal AA (2008). Plant genotype shapes ant-aphid interactions: Implications for community structure and indirect plant defense. Am. Nat..

[66] Bingham RA, Agrawal AA (2010). Specificity and trade-offs in the induced plant defence of common milkweed *Asclepias syriaca* to two lepidopteran herbivores. J. Ecol..

[67] Vannette RL, Hunter MD (2011). Genetic variation in expression of defense phenotype may mediate evolutionary adaptation of *Asclepias syriaca* to elevated CO_2_. Glob. Chang. Biol..

[68] Woods EC, Hastings AP, Turley NE, Heard SB, Agrawal AA (2012). Adaptive geographical clines in the growth and defense of a native plant. Ecol. Monogr..

[69] Santangelo JS (2022). Global urban environmental change drives adaptation in white clover. Science.

[70] Johnson MTJ, Prashad CM, Lavoignat M, Saini HS (2018). Contrasting the effects of natural selection, genetic drift and gene flow on urban evolution in white clover (*Trifolium repens*). Proc. Biol. Sci..

[71] Rivkin LR, Nhan VJ, Weis AE, Johnson MTJ (2020). Variation in pollinator-mediated plant reproduction across an urbanization gradient. Oecologia.

[72] Czúni, L., Lipovits, Á. & Seress, G. Estimation of urbanization using visual features of satellite images. in *Proceedings of the AGILE’2012 International Conference on Geographic Information Science, Avignon, France* 24–27 (2012).

[73] Seress G, Lipovits Á, Bókony V, Czúni L (2014). Quantifying the urban gradient: A practical method for broad measurements. Landsc. Urban Plan..

[74] Lipovits, Á., Czúni, L. & Seress, G. A tool for quantifying the urban gradient. in Athens: ATINER’S Conference Paper Series, No: PLA2015-1709 (2015).

[75] Johnson MTJ, Bertrand JA, Turcotte MM (2016). Precision and accuracy in quantifying herbivory. Ecol. Entomol..

[76] Agrawal AA, Van Zandt PA (2003). Ecological play in the coevolutionary theatre: Genetic and environmental determinants of attack by a specialist weevil on milkweed. J. Ecol..

[77] Petschenka G, Züst T, Hastings AP, Agrawal AA, Jander G, Jez J (2023). Quantification of plant cardenolides by HPLC, measurement of Na+/K+-ATPase inhibition activity, and characterization of target enzymes. Methods in Enzymology.

[78] R Core Team. R: A Language and Environment for Statistical Computing. https://www.R-project.org (2021).

[79] Brooks M (2017). GlmmTMB balances speed and flexibility among packages for zero-inflated generalized linear mixed modeling. R J..

[80] Bates D, Mächler M, Bolker B, Walker S (2015). Fitting linear mixed-effects models using lme4. J. Stat. Softw..

[81] Spitze K (1993). Population structure in *Daphnia obtusa*: Quantitative genetic and allozymic variation. Genetics.

[82] Houle D (1992). Comparing evolvability and variability of quantitative traits. Genetics.

[83] Kuznetsova A, Brockhoff PB, Christensen RHB (2017). lmerTest package: Tests in linear mixed effects models. J. Stat. Softw..

[84] Self SG, Liang K-Y (1987). Asymptotic properties of maximum likelihood estimators and likelihood ratio tests under nonstandard conditions. J. Am. Stat. Assoc..

[85] Halekoh U, Højsgaard S (2014). A Kenward-Roger approximation and parametric bootstrap methods for tests in linear mixed models—The R package pbkrtest. J. Stat. Softw..

[86] Hartig, F. DHARMa: residual diagnostics for hierarchical (multi-level/mixed) regression models. *R package version 0.4.3* (2021).

[87] Lüdecke D, Ben-Shachar M, Patil I, Waggoner P, Makowski D (2021). Performance: An R package for assessment, comparison and testing of statistical models. J. Open Source Softw..

[88] Mullahy J (1986). Specification and testing of some modified count data models. J. Econom..

[89] Clopper CJ, Pearson ES (1934). The use of confidence or fiducial limits illustrated in the case of the binomial. Biometrika.

[90] Zar JH (1996). Biostatistical Analysis.

[91] Fox J, Weisberg S (2019). An R Companion to Applied Regression.

[92] Langsrud Ø (2003). ANOVA for unbalanced data: Use Type II instead of Type III sums of squares. Stat. Comput..

[93] Wang Y, Naumann U, Wright ST, Warton DI (2012). mvabund - an R package for model-based analysis of multivariate abundance data. Methods Ecol. Evol..

[94] Bozdogan H (1987). Model selection and Akaike’s Information Criterion (AIC): The general theory and its analytical extensions. Psychometrika.

[95] Agrawal AA (2004). Resistance and susceptibility of milkweed: Competition, root herbivory, and plant genetic variation. Ecology.

[96] Agrawal AA (2015). Evolution of plant growth and defense in a continental introduction. Am. Nat..

[97] Potts AS, Hunter MD (2021). Unraveling the roles of genotype and environment in the expression of plant defense phenotypes. Ecol. Evol..

[98] Fukano Y, Guo W, Uchida K, Tachiki Y (2020). Contemporary adaptive divergence of plant competitive traits in urban and rural populations and its implication for weed management. J. Ecol..

[99] Canada. Department of Agriculture. *Census of Canada. 1870–71, Volume 1*. (1873).

[100] Holsinger KE (2000). Reproductive systems and evolution in vascular plants. Proc. Natl. Acad. Sci. U. S. A..

[101] Barrett SCH (2015). Influences of clonality on plant sexual reproduction. Proc. Natl. Acad. Sci. U. S. A..

[102] Kephart SR (1981). Breeding Systems in *Asclepias incarnata* L.*, A. syriaca* L.*,* and *A. verticillata* L.. Am. J. Bot..

[103] Beekman M, Ratnieks FLW (2000). Long-range foraging by the honey-bee, *Apis mellifera* L.. Funct. Ecol..

[104] Walther-Hellwig K, Frankl R (2000). Foraging Distances of *Bombus muscorum, Bombus lapidarius*, and *Bombus terrestris* (Hymenoptera, Apidae). J. Insect Behav..

[105] Moll RJ (2019). What does urbanization actually mean? A framework for urban metrics in wildlife research. J. Appl. Ecol..

[106] Cadenasso ML, Pickett STA, Schwarz K (2007). Spatial heterogeneity in urban ecosystems: Reconceptualizing land cover and a framework for classification. Front. Ecol. Environ..

[107] Ramalho CE, Hobbs RJ (2012). Time for a change: Dynamic urban ecology. Trends Ecol. Evol..

[108] Tewksbury JJ (2002). Corridors affect plants, animals, and their interactions in fragmented landscapes. Proc. Natl. Acad. Sci. U. S. A..

[109] Townsend PA, Levey DJ (2005). An experimental test of whether habitat corridors affect pollen transfer. Ecology.

[110] Damschen EI (2014). How fragmentation and corridors affect wind dynamics and seed dispersal in open habitats. Proc. Natl. Acad. Sci. U. S. A..

[111] Saarikivi J, Knopp T, Granroth A, Merilä J (2013). The role of golf courses in maintaining genetic connectivity between common frog (*Rana temporaria*) populations in an urban setting. Conserv. Genet..

[112] Gortat T (2015). Anthropopressure gradients and the population genetic structure of *Apodemus agrarius*. Conserv. Genet..

[113] Braaker S, Kormann U, Bontadina F, Obrist MK (2017). Prediction of genetic connectivity in urban ecosystems by combining detailed movement data, genetic data and multi-path modelling. Landsc. Urban Plan..

[114] Angold PG (2006). Biodiversity in urban habitat patches. Sci. Total Environ..

[115] Blanchet É (2015). Multivariate analysis of polyploid data reveals the role of railways in the spread of the invasive South African Ragwort (*Senecio inaequidens*). Conserv. Genet..

[116] Beninde J, Feldmeier S, Veith M, Hochkirch A (2018). Admixture of hybrid swarms of native and introduced lizards in cities is determined by the cityscape structure and invasion history. Proc. Biol. Sci..

[117] Kupfer JA, Malanson GP, Franklin SB (2006). Not seeing the ocean for the islands: the mediating influence of matrix-based processes on forest fragmentation effects. Glob. Ecol. Biogeogr..

[118] Travers E, Härdtle W, Matthies D (2021). Corridors as a tool for linking habitats—Shortcomings and perspectives for plant conservation. J. Nat. Conserv..

