# Supplementary material for: Urbanization and a green corridor do not impact genetic divergence in common milkweed (Asclepias syriaca L.)
Source: Sci Rep. 2023 Nov 22;13:20437. doi: 10.1038/s41598-023-47524-8 (PMC10665382; doi:10.1038/s41598-023-47524-8)
Supplement: Supplementary file 1 — Supplementary Information 1. [file 41598_2023_47524_MOESM1_ESM.docx]

**Online supplementary for**: Urbanization and a green corridor do not impact genetic divergence in common milkweed (*Asclepias syriaca*)

**Authors**: Sophie T. Breitbart (1,2,3), Anurag A. Agrawal (4,5), Helene H. Wagner (1,2,3), Marc T.J. Johnson (1,2,3)

1. Department of Ecology and Evolutionary Biology

University of Toronto

25 Willcocks Street

Toronto, Ontario

Canada M5S 3B2

1. Department of Biology

University of Toronto Mississauga

3359 Mississauga Road

Mississauga, ON

Canada L5L 1C6

1. Centre for Urban Environments

University of Toronto Mississauga

3359 Mississauga Road

Mississauga, ON

Canada L5L 1C6

1. Department of Ecology & Evolutionary Biology

Cornell University

E145 Corson Hall

Ithaca, NY

USA 14853

1. Department of Entomology

Cornell University

2126 Comstock Hall

Ithaca, NY

USA 14853

**Corresponding author**: Sophie Breitbart (https://orcid.org/0000-0001-9641-9786) ([sophie.breitbart@gmail.com](mailto:sophie.breitbart@gmail.com))

Contents:

- Supplementary text S1
- Supplementary tables and figures:
  - Supplementary figures: Figures S1-S23
  - Supplementary tables: Tables S1-S14

## **Text S1**

***Common garden experiment***

Full-sibling seeds were cleaned with 5% bleach, mechanically scarified with a razor, vernalized via refrigeration at 4°C for a week, and incubated at 30°C for 3-4 days until germination occurred. Upon germination, two full-sib seeds were planted in the same pot with potting soil (Pro-Mix LP15, Sun Gro Horticulture) and Nutricote 14:13:13 (N:P:K) slow release fertilizer (Plant Products Inc., Ancaster, Canada). Pots were placed in a growth chamber set to 27°C daytime/25°C nighttime temperatures with 14h light:10h dark and 750 µmol/m²/s of light with 50% humidity. Plants were watered daily and randomized one week after planting. All pots were thinned to one seedling after two weeks. If both seeds sprouted two weeks after planting, one seedling was wholly removed.

Once brought to KSR, pots were sunk into the ground within a hole cut in the fabric. Seedlings were watered immediately after transplanting as well as once during the first week after transplanting and four weeks after transplanting due to unusually hot and dry conditions. Vegetated laneways next to rows were routinely mowed throughout the growing season (May-September). Pots were weeded annually and otherwise grown under natural conditions for the remainder of the experiment. Landscaping fabric was replaced in 2020 (Hanes Geo Components, Winston-Salem, NC, USA) due to degradation.

## ***Trait measurements***

We generated population-level estimates of leaf cardenolide concentrations by collecting the leaf used to assess latex exudation and its opposite, placing leaves on dry ice, and storing at -80°C until freeze-dried. We cut tissue from each replicate, excluding the mid-rib, and pooled the samples by population so that each population’s vial contained 50 mg of tissue from 5 replicates per family, and 5 families per population except when mortality prevented collection from all plants within a family or population. For instance, we collected 2 mg/replicate if a population contained the full 25 replicates; otherwise, we collected >2mg/replicate for up to 10 mg/family for populations containing <25 replicates.

To assess specific leaf area (SLA) and leaf dry matter content (LDMC), we collected the youngest fully expanded intact leaf, placed it in a coin envelope on dry ice, and saturated the leaves in water for 13 h. Leaves were dabbed with a paper towel, photographed with a ruler for scale, weighed for wet mass, and then dried at 60°C for 48 hours, after which leaves were re-weighed for dry mass. Leaf length, width, and area were measured with ImageJ^1^.

### ***Statistical analyses***

#### *Genetic differentiation between a green corridor & urban matrix (Q3)*

For Questions 2 and 3, we initially fitted multi-year models with the addition of Year as a fixed effect. We fitted 1-year models by removing the effect of year from the multi-year models and restricting the data to the last year of sampling, then compared the significance of each fixed effect between analogous multi-year and 1-year models. When we tested for the consistency of urbanization and green corridors across all years of data collection, we found that these effects were qualitatively identical to those reported in Tables [2-3](file:////l) 85% of the time (Supplementary Table 3).

## **Figures**


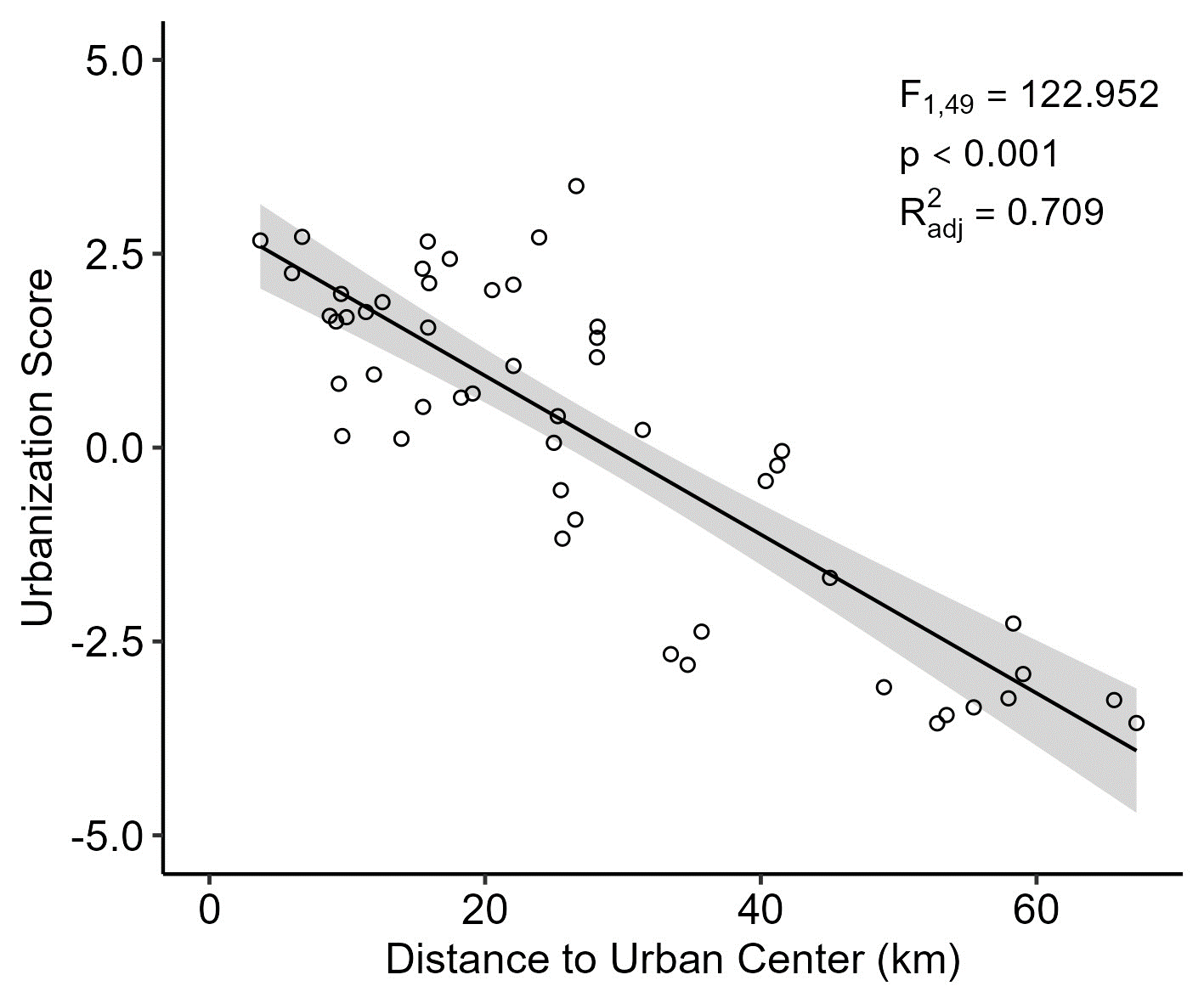


**Supplementary Figure 1.** The correlation between both metrics of urbanization: Distance to Urban Center and Urbanization Score. A regression line with a 95% confidence envelope and points representing populations are shown for the general linear model.


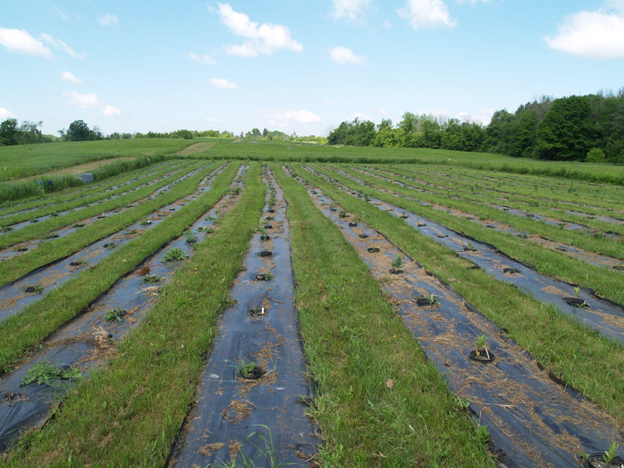


**Supplementary Figure 2.** Common garden experiment setup. Photo credit: Sophie Breitbart

**
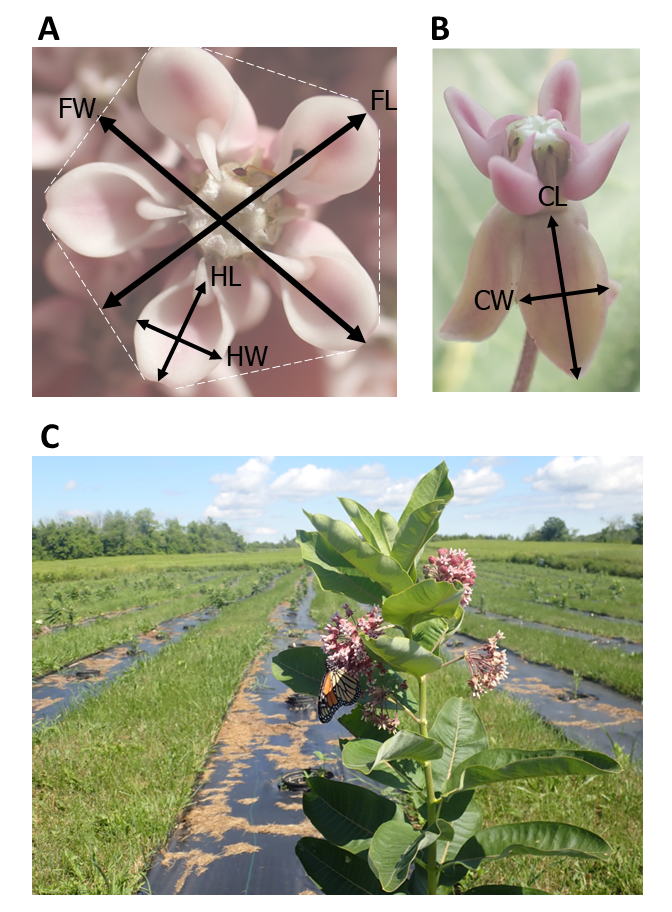
**

**Supplementary Figure 3.** A) Example of hood length (HL), hood width (HW), flower length (FL), and flower width (FW) measurements. B) Example of corolla length (CL) and corolla width (CW) measurements. All six measurements were averaged to calculate mean size per flower. C) A flowering *A. syriaca* ramet within the common garden. Photo credit: Sophie Breitbart

**
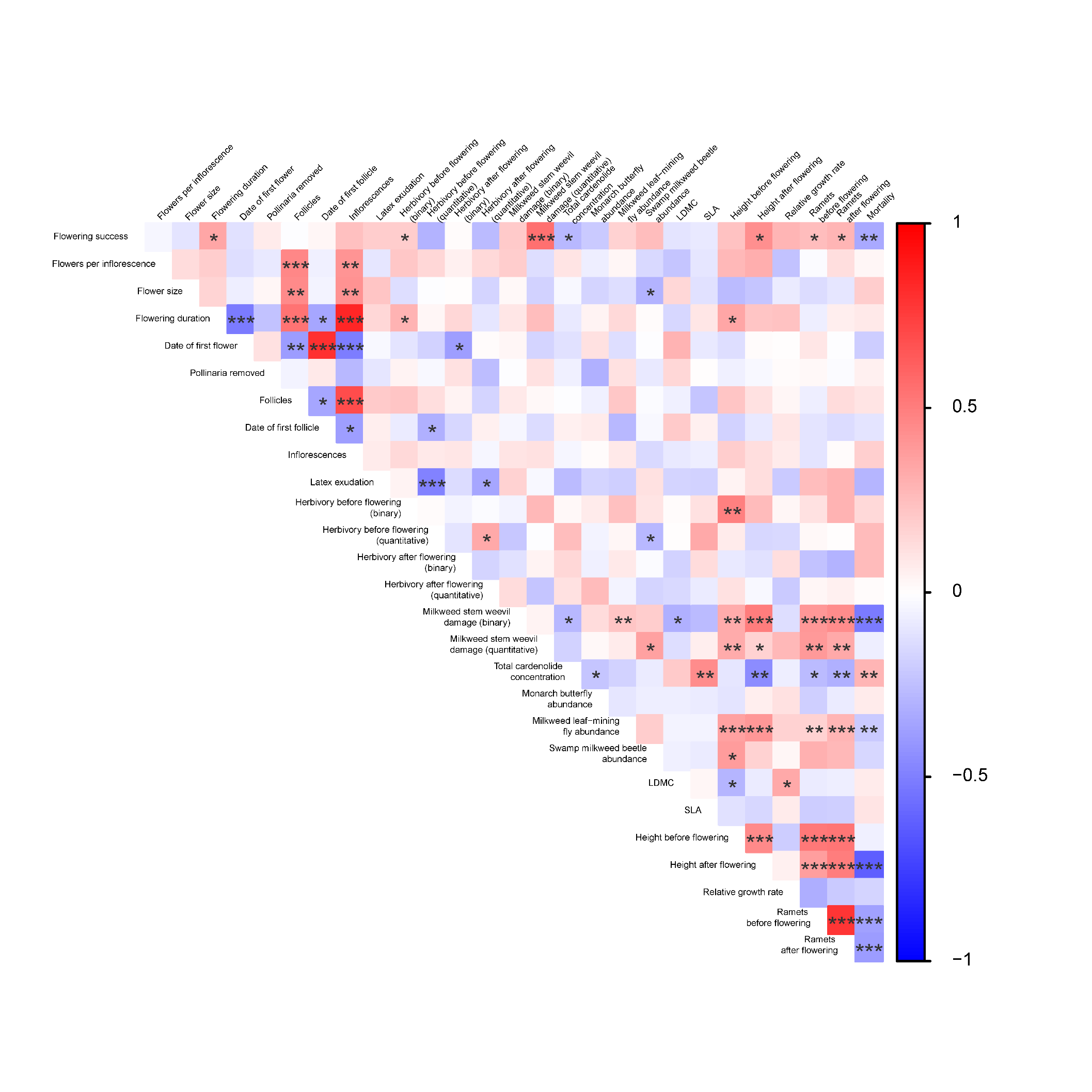
**

**Supplementary Figure 4.** Correlation matrix of traits associated with plant reproduction, defense, growth, and herbivore abundance. Pearson correlation coefficients were calculated using population-level estimates. Colors represent correlation coefficients while asterisks represent levels of p value significance levels (* p ≤ 0.05; ** p ≤ 0.01; *** p ≤ 0.001).


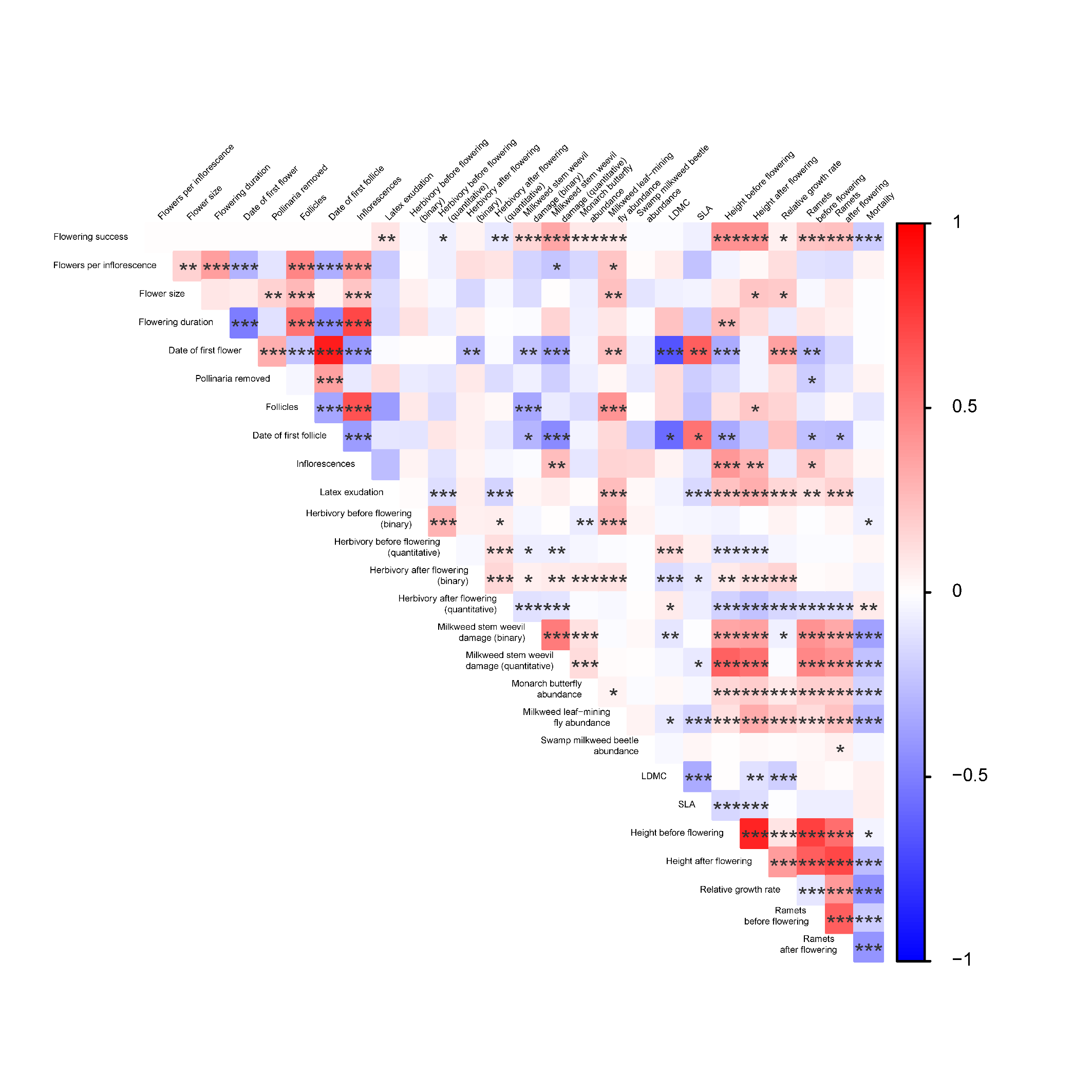


**Supplementary Figure 5.** Correlation matrix of traits associated with plant reproduction, defense, growth, and herbivore abundance. Pearson correlation coefficients were calculated using individual-level measurements. As reproductive traits necessitated a flowering success value of 1, correlations were not calculated between flowering success and flowers per inflorescence, flower size, flowering duration, date of first flower, follicles, date of first follicle, and inflorescences. Cardenolide concentration was excluded because this was estimated at the population level only. Colors represent correlation coefficients while asterisks represent levels of p value significance levels (* p ≤ 0.05; ** p ≤ 0.01; *** p ≤ 0.001).

**
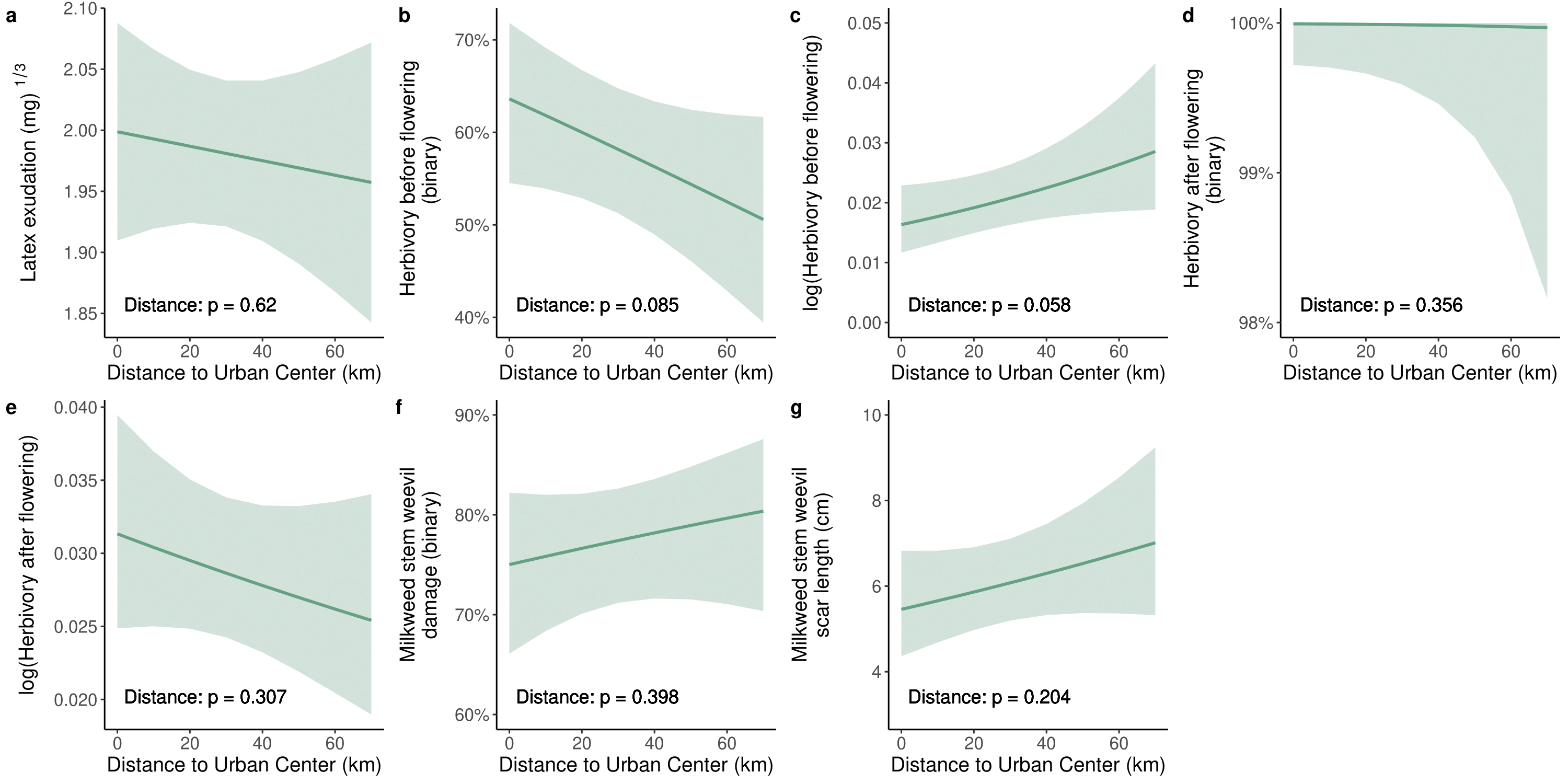
Supplementary Figure 6.** The effect of urbanization on plant defense/damage traits when urbanization was quantified by distance from the urban center. Regression lines with a 95% confidence envelope for the mean response are shown for general and generalized linear mixed effects models.


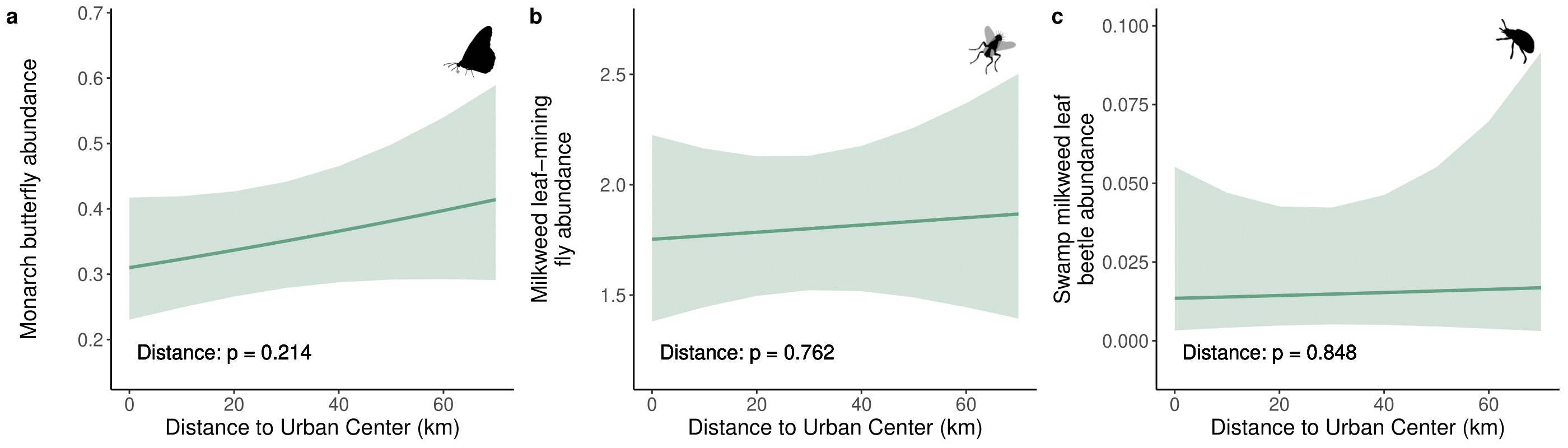


**Supplementary Figure 7.** The effect of urbanization on herbivore abundance when urbanization was quantified by distance from the urban center. Regression lines with a 95% confidence envelope for the mean response are shown for generalized linear mixed effects models.


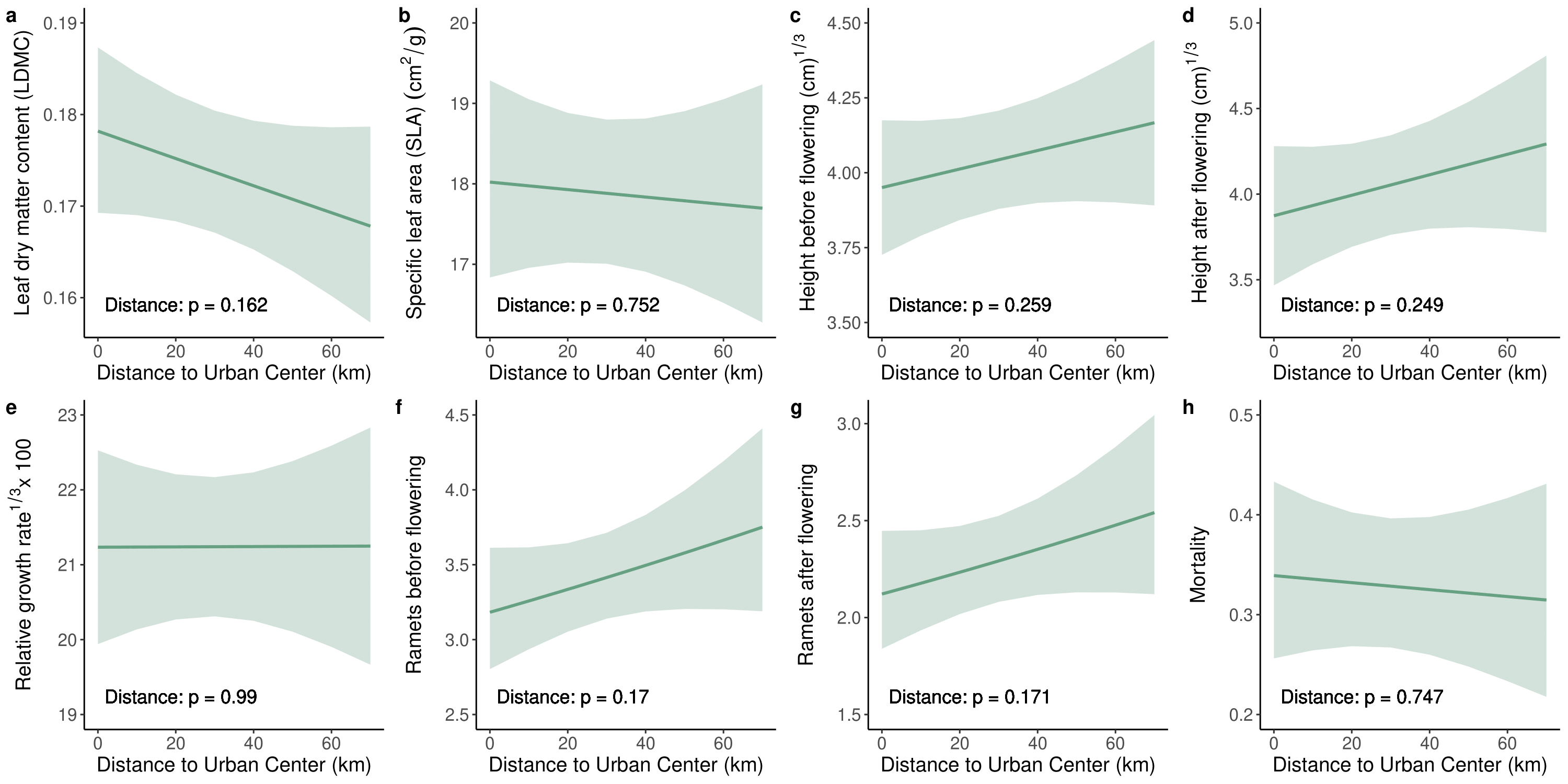


**Supplementary Figure 8.** The effect of urbanization on plant growth traits when urbanization was quantified by distance from the urban center. Regression lines with a 95% confidence envelope for the mean response are shown for general and generalized linear mixed effects models.


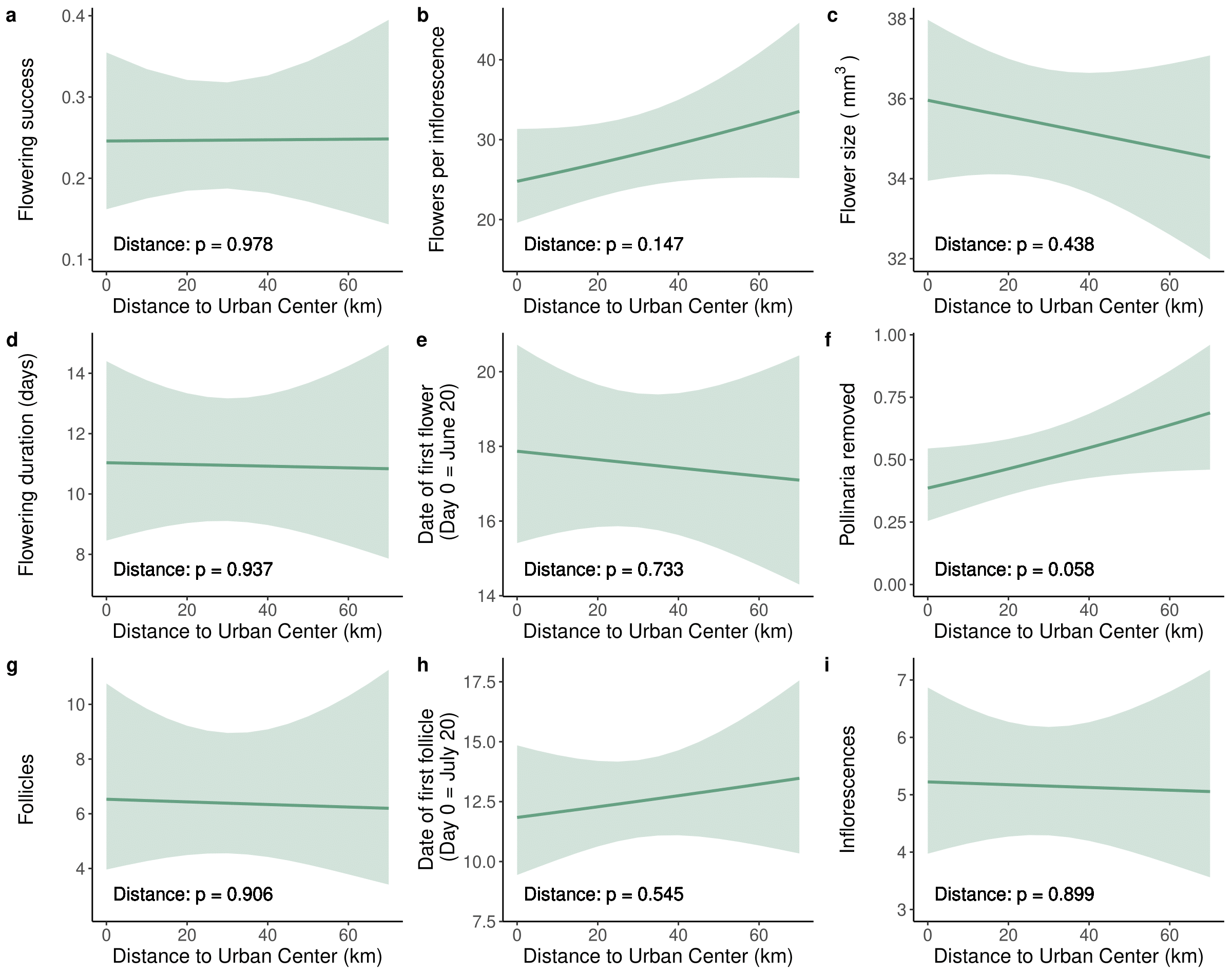


**Supplementary Figure 9.** The effect of urbanization on plant reproduction traits when urbanization was quantified by distance from the urban center. Regression lines with a 95% confidence envelope for the mean response are shown for general and generalized linear mixed effects models.


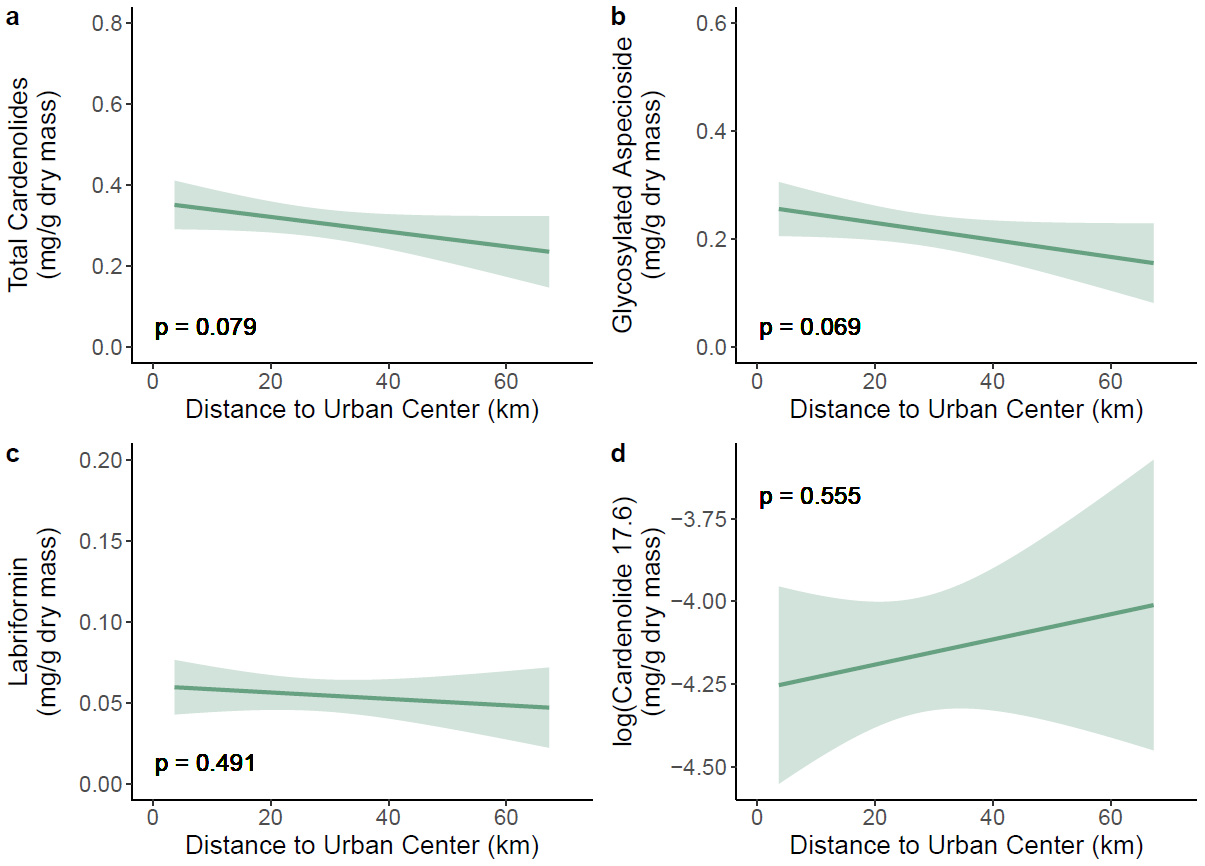


**Supplementary Figure 10.** The effect of urbanization on cardenolides when urbanization was quantified by distance from the urban center. Regression lines with a 95% confidence envelope for the mean response are shown for general linear mixed effects models. Cardenolide 17.6 is an unidentified cardenolide with a retention time of 17.6 minutes.

**
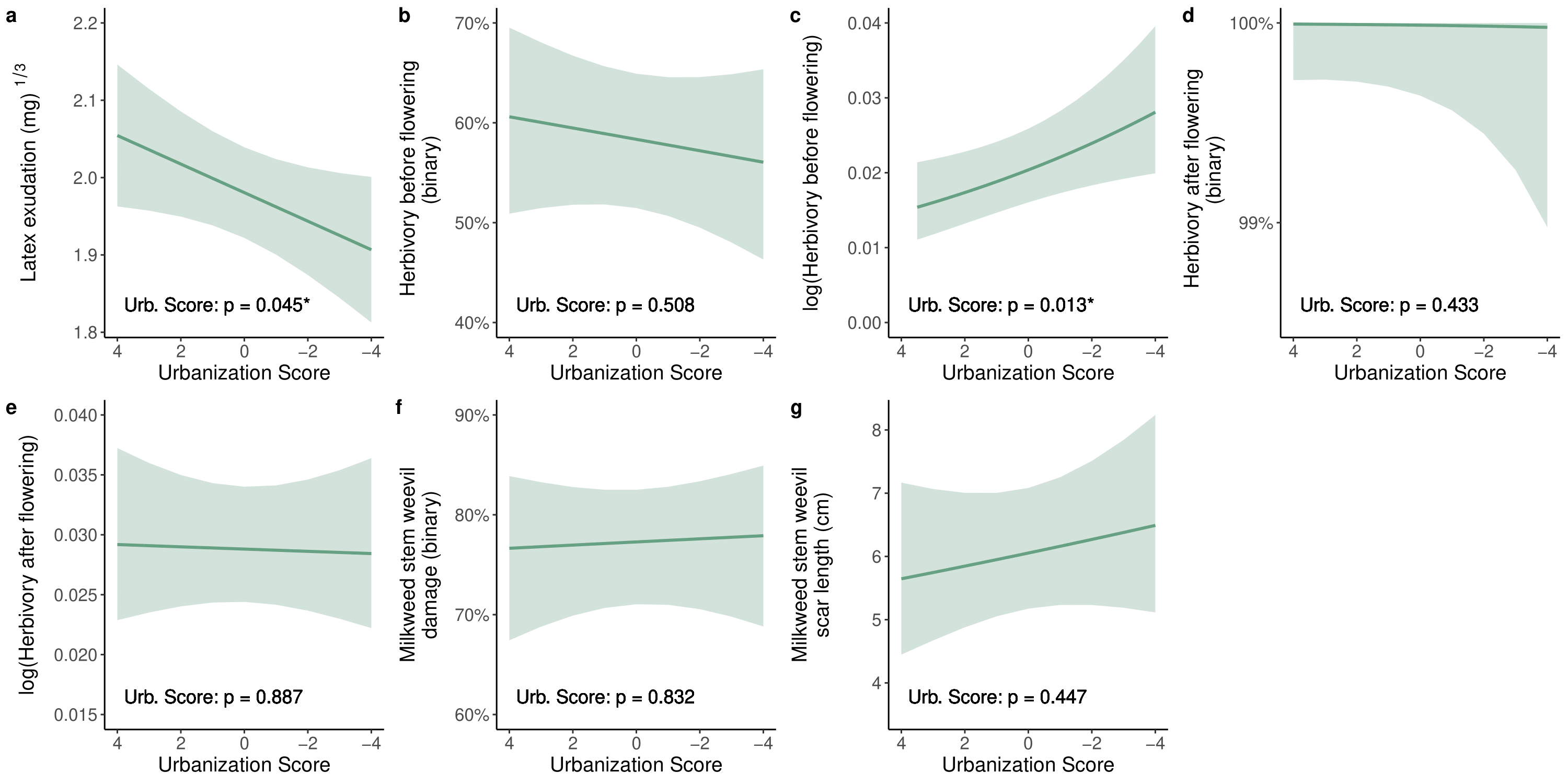
Supplementary Figure 11.** The effect of urbanization on plant defense/damage traits when urbanization was quantified by urbanization score. Regression lines with a 95% confidence envelope for the mean response are shown for general and generalized linear mixed effects models.


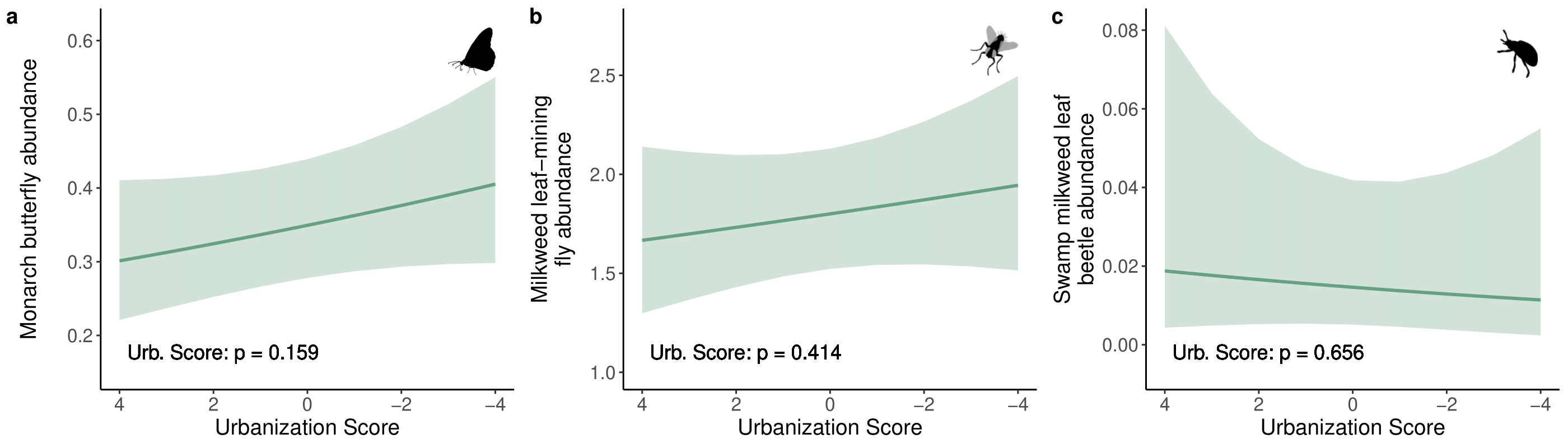


**Supplementary Figure 12.** The effect of urbanization on herbivore abundance when urbanization was quantified by urbanization score. Regression lines with a 95% confidence envelope for the mean response are shown for generalized linear mixed effects models.


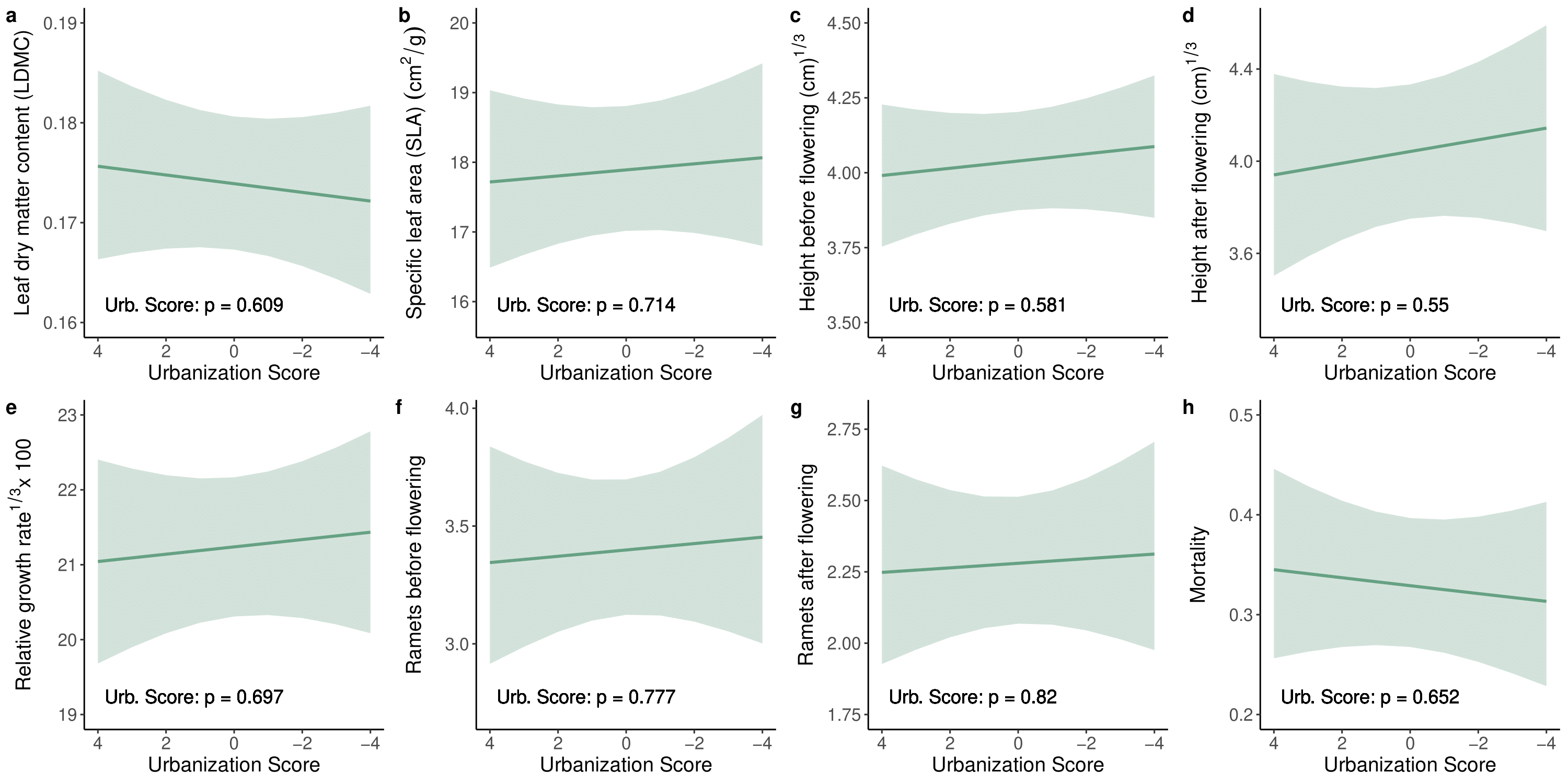


**Supplementary Figure 13.** The effect of urbanization on plant growth traits when urbanization was quantified by urbanization score. Regression lines with a 95% confidence envelope for the mean response are shown for general and generalized linear mixed effects models.


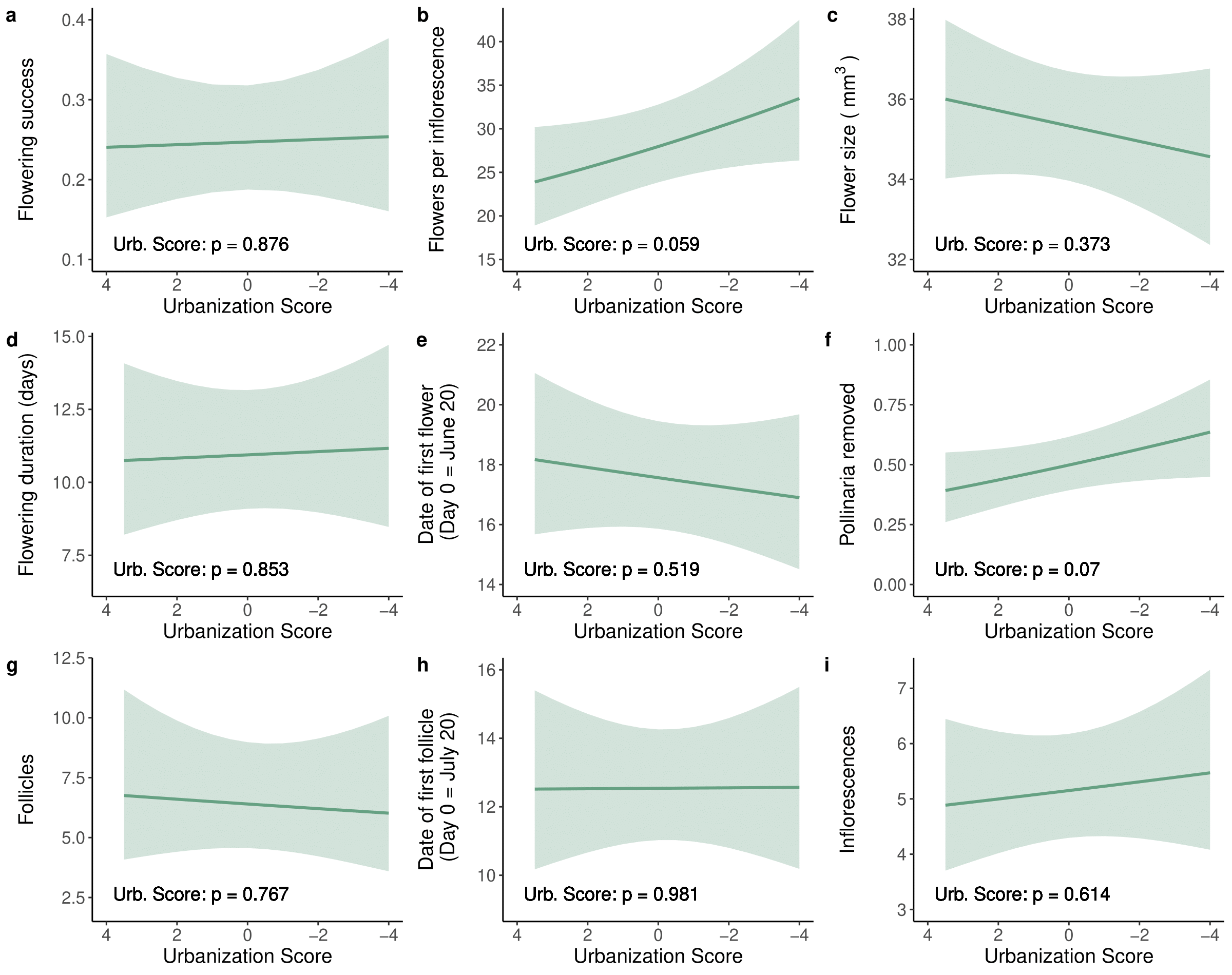


**Supplementary Figure 14.** The effect of urbanization on plant reproduction traits when urbanization was quantified by urbanization score. Regression lines with a 95% confidence envelope for the mean response are shown for general and generalized linear mixed effects models.


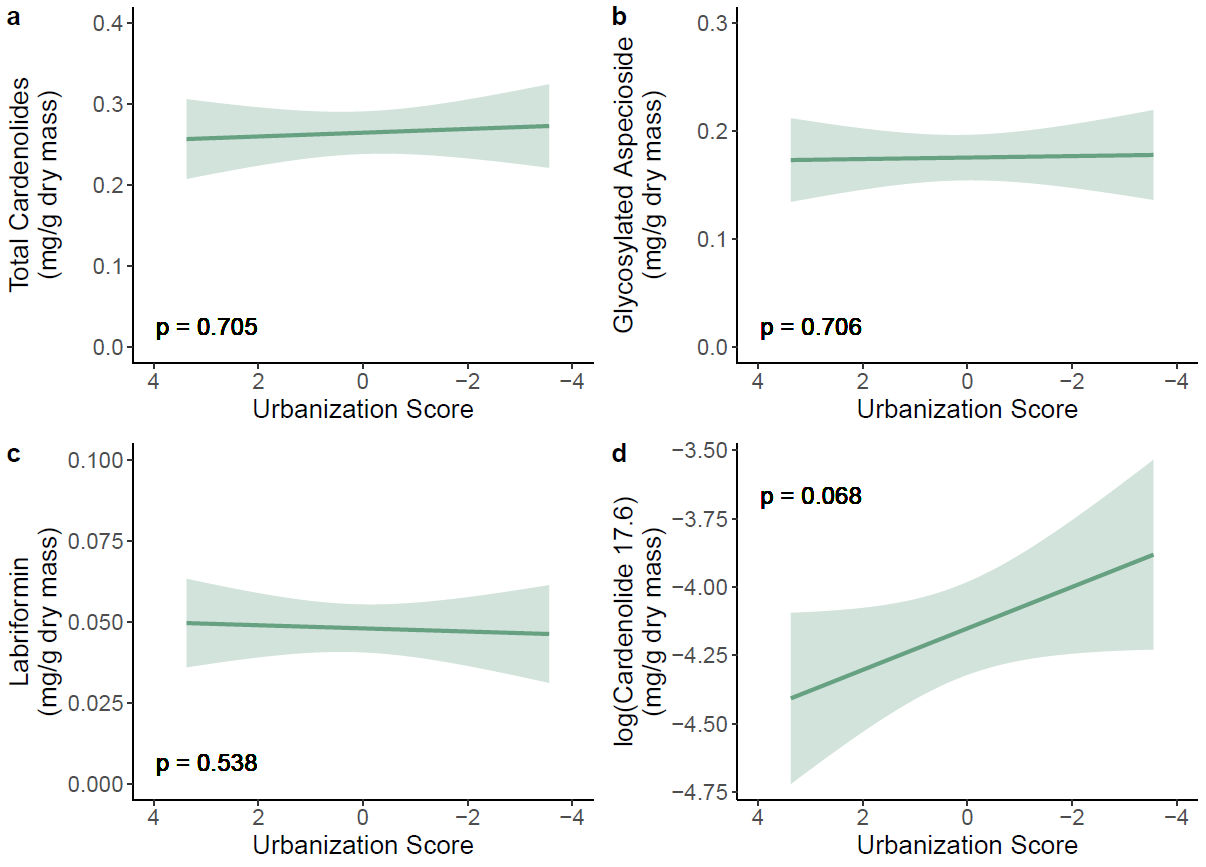


**Supplementary Figure 15.** The effect of urbanization on cardenolides when urbanization was quantified by urbanization score. Regression lines with a 95% confidence envelope for the mean response are shown for general linear mixed effects models. Cardenolide 17.6 is an unidentified cardenolide with a retention time of 17.6 minutes.

**
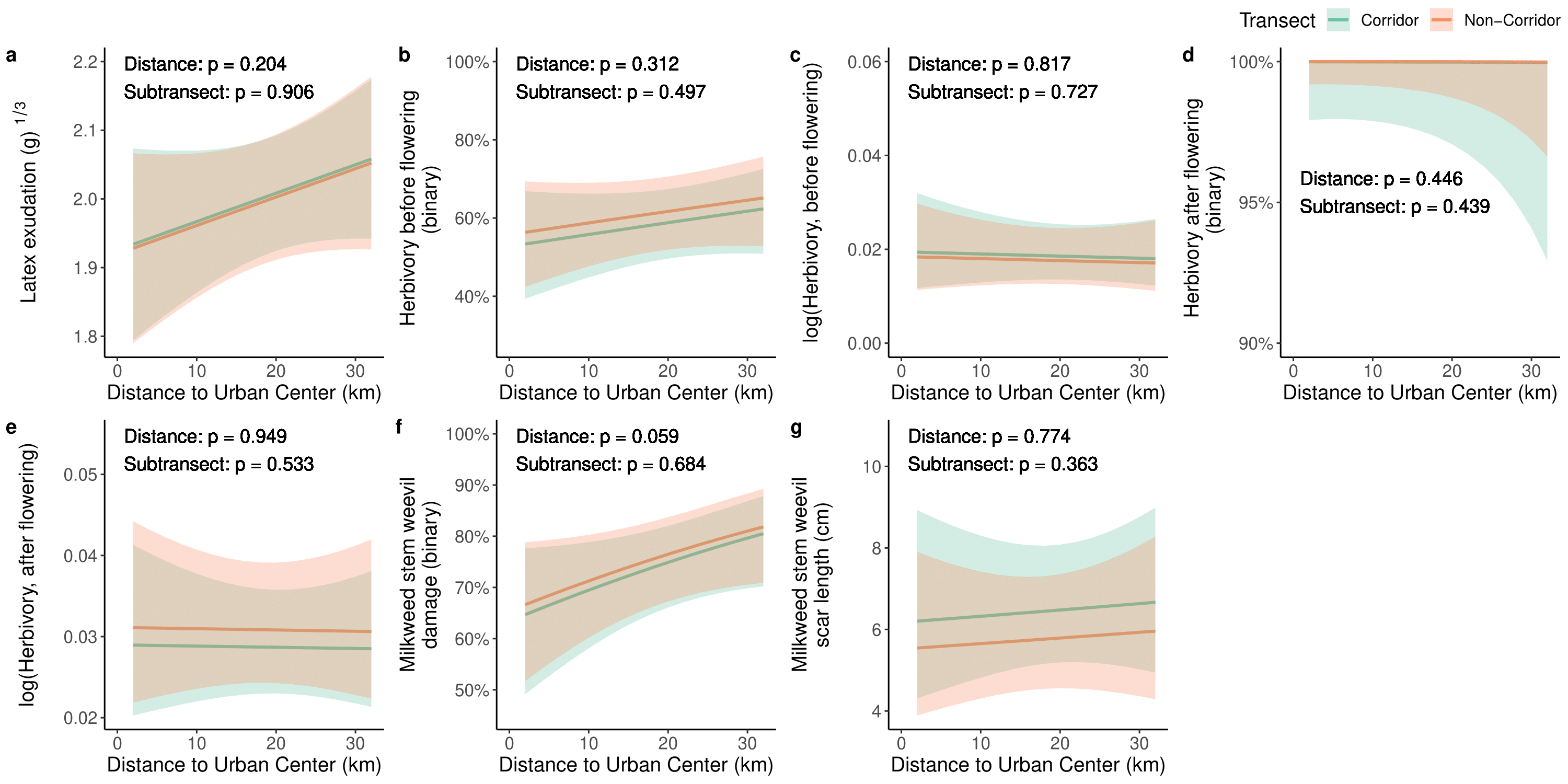
Supplementary Figure 16.** The effects of urbanization and proximity to a green corridor on plant defense/damage traits when urbanization was quantified by distance from the urban center. Regression lines with a 95% confidence envelope for the mean response, separately for each subtransect, are shown for general and generalized linear mixed effects models.


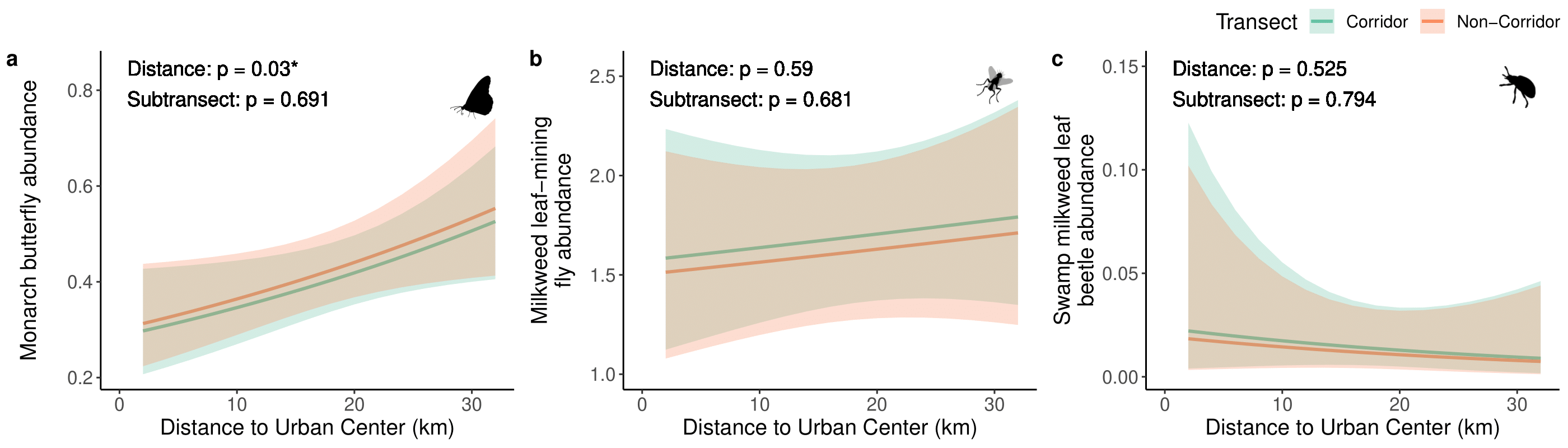


**Supplementary Figure 17.** The effects of urbanization and proximity to a green corridor on herbivore abundance when urbanization was quantified by distance from the urban center. Regression lines with a 95% confidence envelope for the mean response, separately for each subtransect, are shown for generalized linear mixed effects models.


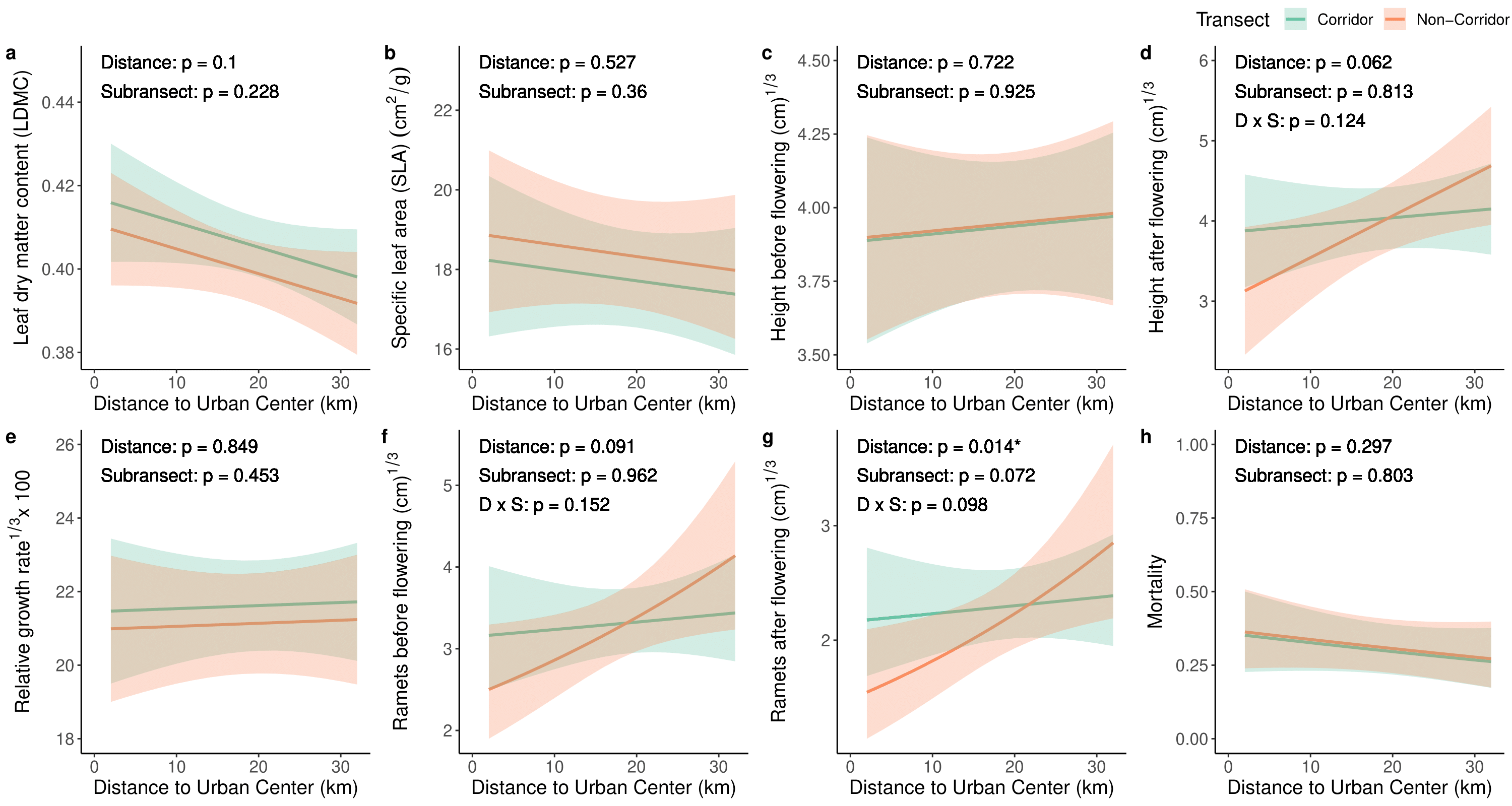


**Supplementary Figure 18.** The effects of urbanization and proximity to a green corridor on plant growth traits when urbanization was quantified by distance from the urban center. Regression lines with a 95% confidence envelope for the mean response, separately for each subtransect, are shown for general and generalized linear mixed effects models.


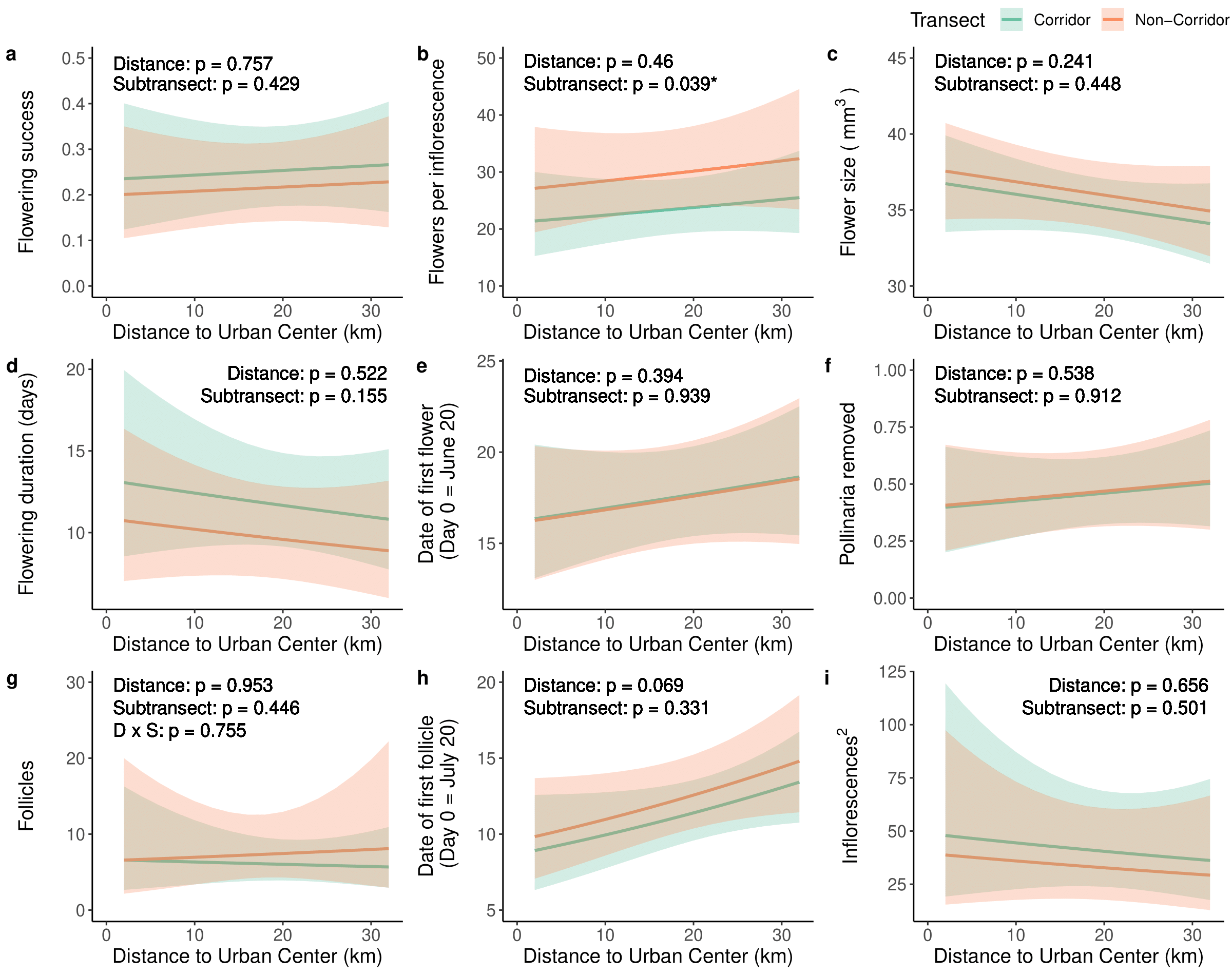


**Supplementary Figure 19.** The effects of urbanization and proximity to a green corridor on plant reproduction traits when urbanization was quantified by distance from the urban center. Regression lines with a 95% confidence envelope for the mean response, separately for each subtransect, are shown for general and generalized linear mixed effects models.

**
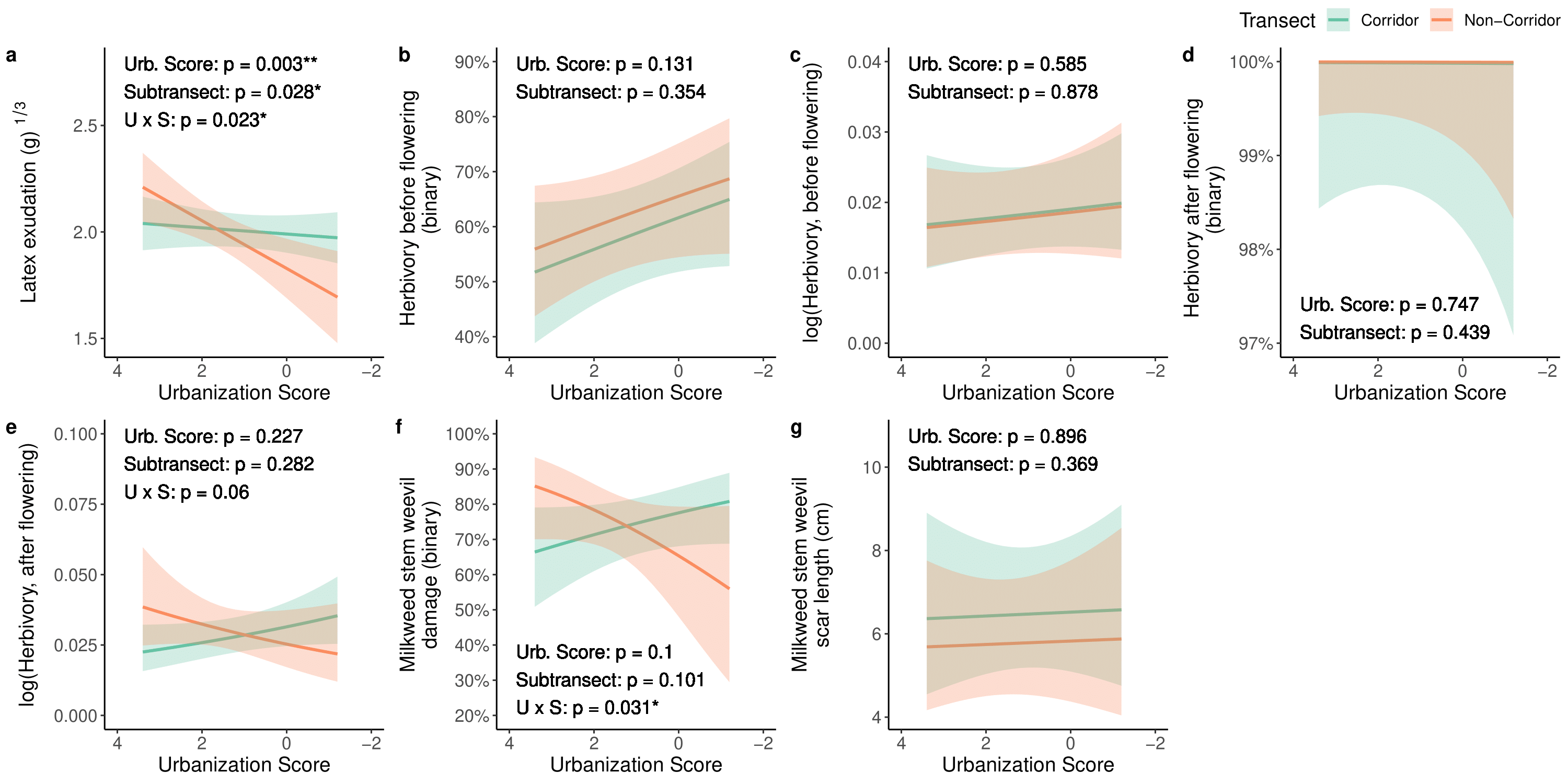
Supplementary Figure 20.** The effects of urbanization and proximity to a green corridor on plant defense/damage traits when urbanization was quantified by urbanization score. Regression lines with a 95% confidence envelope for the mean response, separately for each subtransect, are shown for general and generalized linear mixed effects models.


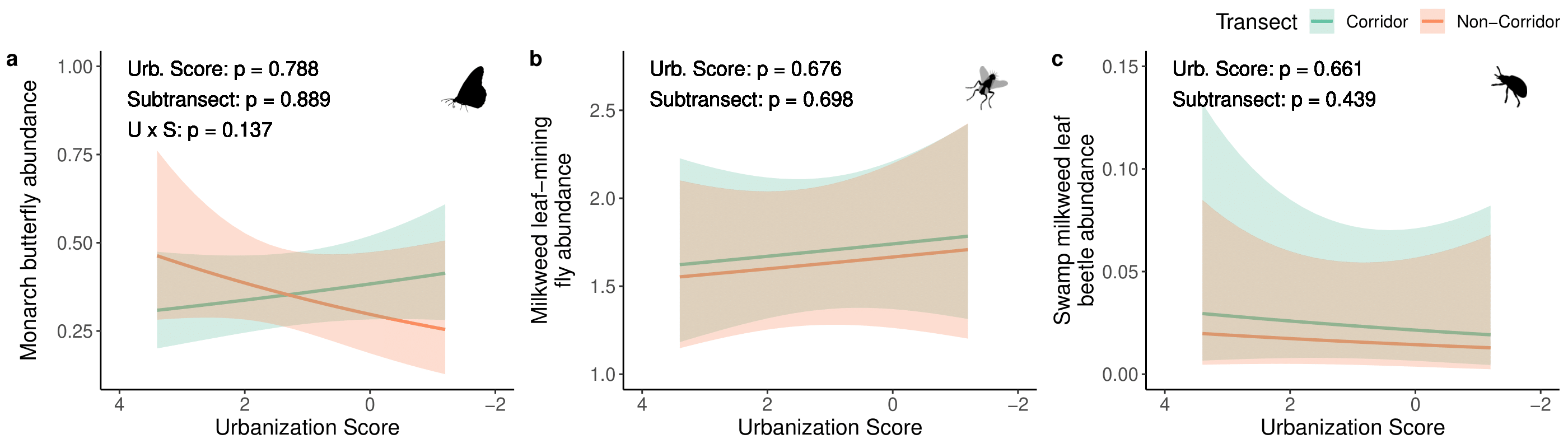


**Supplementary Figure 21.** The effects of urbanization and proximity to a green corridor on herbivore abundance when urbanization was quantified by urbanization score. Regression lines with a 95% confidence envelope for the mean response, separately for each subtransect, are shown for generalized linear mixed effects models.


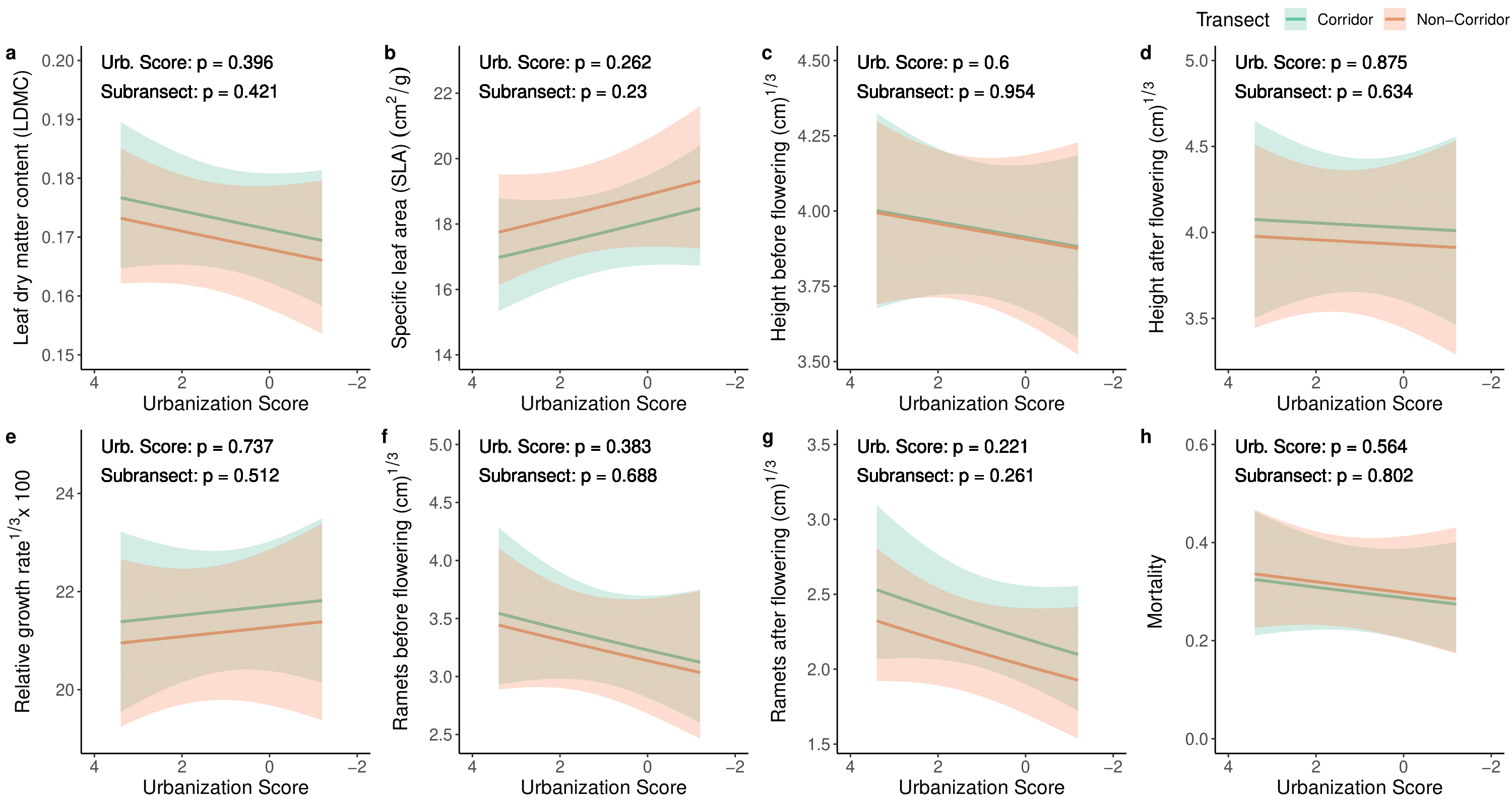


**Supplementary Figure 22.** The effects of urbanization and proximity to a green corridor on plant growth traits when urbanization was quantified by urbanization score. Regression lines with a 95% confidence envelope for the mean response, separately for each subtransect, are shown for general and generalized linear mixed effects models.


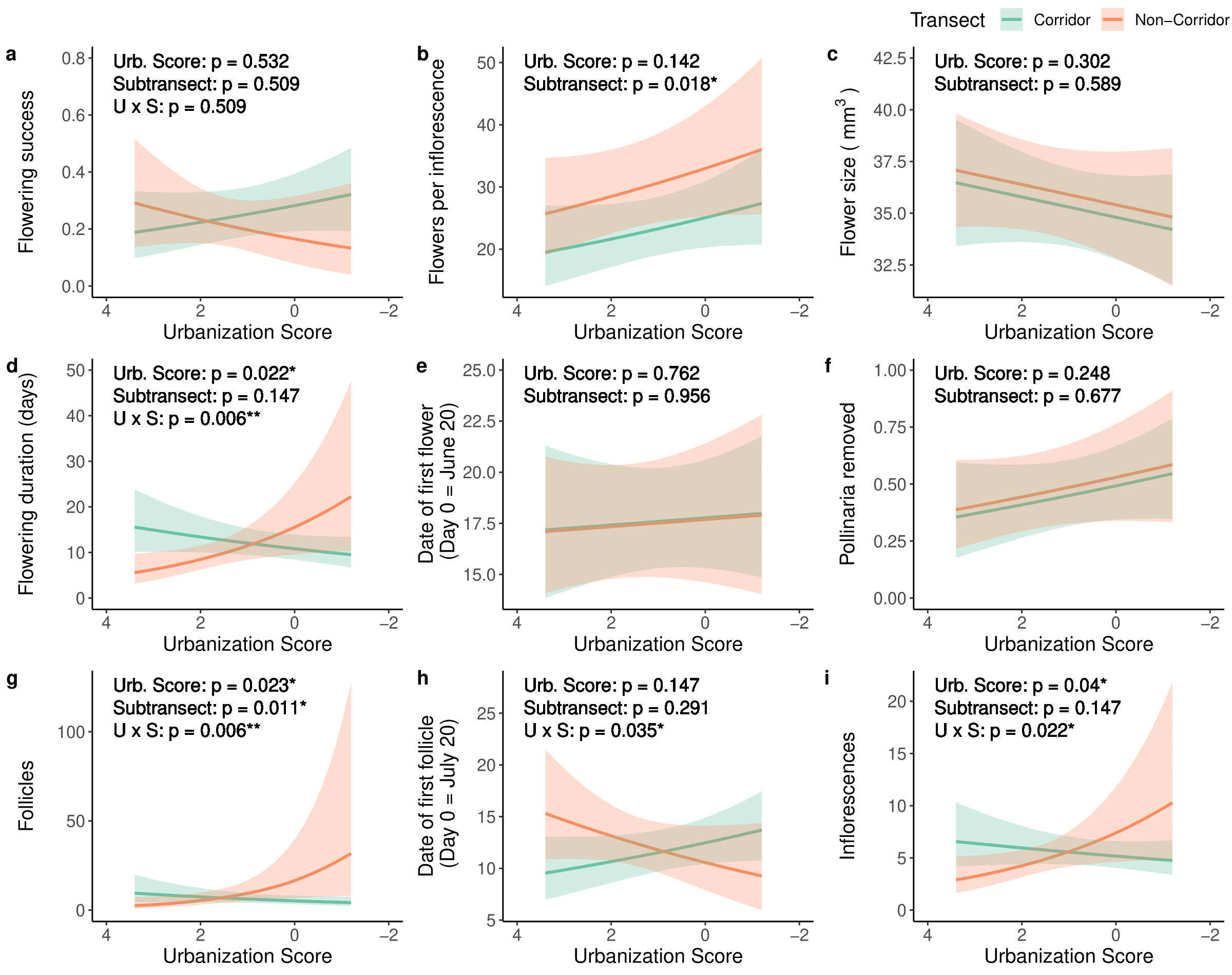


**Supplementary Figure 23.** The effects of urbanization and proximity to a green corridor on plant reproduction traits when urbanization was quantified by urbanization score. Regression lines with a 95% confidence envelope for the mean response, separately for each subtransect, are shown for general and generalized linear mixed effects models.

## **Tables**

|  | Distance | | Urbanization Score | |
| --- | --- | --- | --- | --- |
|  | All Populations | Urban Populations | All Populations | Urban Populations |
| Height before flowering | x^1/3^ | x^1/3^ | x^1/3^ | x^1/3^ |
| Height after flowering | x^1/3^ | x^1/3^ | x^1/3^ | x^1/3^ |
| LDMC | x^1/2^ | x^1/2^ | x^1/2^ | x^1/2^ |
| Mortality | - | - | - | - |
| Ramets before flowering | - | - | - | - |
| Ramets after flowering | - | - | - | - |
| Relative growth rate | x^1/3^ × 100 | x^1/3^ × 100 | x^1/3^ × 100 | x^1/3^ × 100 |
| SLA | log(x + 1) | log(x + 1) | log(x + 1) | log(x + 1) |
| Monarch butterfly abundance | - | - | - | - |
| Swamp milkweed beetle abundance | - | - | - | - |
| Milkweed leaf-mining fly abundance | - | - | - | - |
| Herbivory before flowering (binary) | - | - | - | - |
| Herbivory before flowering (quantitative) | log(x) | log(x) | log(x) | log(x) |
| Herbivory after flowering (binary) | - | - | - | - |
| Herbivory after flowering (quantitative) | log(x) | log(x) | log(x) | log(x) |
| Latex exudation | x^1/3^ | x^1/3^ | x^1/3^ | x^1/3^ |
| Milkweed stem weevil damage (binary) | - | - | - | - |
| Milkweed stem weevil damage (quantitative) | log(x) | log(x) | log(x) | log(x) |
| Date of first flower | x – 170 | x – 170 | x – 170 | x – 170 |
| Date of first follicle | x – 170 | x – 170 | x – 170 | x – 170 |
| Flower size | - | - | - | - |
| Flowering duration | - | - | - | - |
| Flowering success | - | - | - | - |
| Follicles | - | - | - | - |
| Inflorescences | - | - | x^2^ | - |
| Flowers per inflorescence | - | - | - | - |
| Pollinaria removed | x^1/2^ | x^1/2^ | x^1/2^ | x^1/2^ |
| Cardenolides | - | - | - | - |

**Supplementary Table 1.** Data transformations used on data to improve normality and homogeneity of variance. “X” represents the transformed response variable. A constant of 170 was subtracted from the dates of first flower and follicle due to model convergence issues.

|  | Distance | | Urbanization Score | |
| --- | --- | --- | --- | --- |
|  | All Populations | Urban Populations | All Populations | Urban Populations |
| Height before flowering | - | - | - | - |
| Height after flowering | - | - | - | - |
| LDMC | - | - | - | - |
| Mortality | Binomial | Binomial | Binomial | Binomial |
| Ramets before flowering | Poisson | Poisson | Poisson | Poisson |
| Ramets after flowering | Poisson | Poisson | Poisson | Poisson |
| Relative growth rate | - | - | - | - |
| SLA | - | - | - | - |
| Monarch butterfly abundance | Negative binomial | Negative binomial | Negative binomial | Negative binomial |
| Swamp milkweed beetle abundance | Negative binomial | Negative binomial | Negative binomial | Negative binomial |
| Milkweed leaf-mining fly abundance | Negative binomial | Negative binomial | Negative binomial | Negative binomial |
| Herbivory before flowering (binary) | Binomial | Binomial | Binomial | Binomial |
| Herbivory before flowering (quantitative) | - | - | - | - |
| Herbivory after flowering (binary) | Binomial | Binomial | Binomial | Binomial |
| Herbivory after flowering (quantitative) | - | - | - | - |
| Latex exudation | - | - | - | - |
| Milkweed stem weevil damage (binary) | Binomial | Binomial | Binomial | Binomial |
| Milkweed stem weevil damage (quantitative) | - | - | - | - |
| Date of first flower | Negative binomial | Negative binomial | Negative binomial | Negative binomial |
| Date of first follicle | Poisson | Poisson | Poisson | Poisson |
| Flower size | - | - | - | - |
| Flowering duration | Negative binomial | Negative binomial | Negative binomial | Negative binomial |
| Flowering success | Binomial | Binomial | Binomial | Binomial |
| Follicles | Negative binomial | Negative binomial | Negative binomial | Negative binomial |
| Inflorescences | Negative binomial | Negative binomial | Negative binomial | Negative binomial |
| Flowers per inflorescence | Negative binomial | Negative binomial | Negative binomial | Negative binomial |
| Pollinaria removed | - | - | - | - |
| Cardenolides | - | - | - | - |

**Supplementary Table 2.** Non-Gaussian probability distributions used on all data.

| Sites | ID | Trait | Urbanization | Predictor | p (Multi-year model) | p (1-year model) |
| --- | --- | --- | --- | --- | --- | --- |
| All | 1 | Herbivory before flowering (binary) | Distance | Distance | **0.046** | 0.085 |
|  | 2 | Herbivory before flowering (quantitative) | Urb. score | Urb. score | 0.138 | **0.013** |
|  | 3 | Flowers per inflorescence | Urb. score | Urb. score | **0.037** | 0.059 |
| Urban | 4 | Herbivory before flowering (quantitative) | Urb. score | Urb. score | **0.041** | 0.585 |
|  | 5 | Milkweed stem weevil damage (binary) | Urb. score | U x S | **-** | **0.031** |
|  | 6 | Milkweed stem weevil damage (quantitative) | Distance | Subtransect | **0.034** | 0.363 |
|  | 7 | Milkweed stem weevil damage (quantitative) | Urb. score | Subtransect | **0.030** | 0.369 |
|  | 8 | Date of first follicle | Distance | Distance | **0.011** | 0.069 |
|  | 9 | Flowering duration | Distance | Subtransect | **0.013** | 0.155 |
|  | 10 | Flowering duration | Urb. Score | Urb. score | 0.396 | **0.022** |
|  | 11 | Flowering duration | Urb. Score | U x S | **-** | **0.006** |
|  | 12 | Flowers per inflorescence | Distance | Subtransect | 0.071 | **0.039** |
|  | 13 | Flowers per inflorescence | Urb. score | Urb. score | **0.032** | 0.142 |
|  | 14 | Inflorescences | Urb. score | Subtransect | **0.045** | 0.147 |
|  | 15 | Ramets after flowering | Distance | Distance | 0.090 | **0.014** |
|  | 16 | Mortality | Distance | Distance | **0.015** | 0.297 |

**Supplementary Table 3.** Discrepancies in type III sums-of-squares ANOVA between models including multiple years of data and the last year of data. Shown are the sites included in the models, model comparison ID, trait, urbanization category, model predictor with a p-value that significantly varied among the multi-year vs. 1-year model, p-value for the multi-year model, and p-value for the 1-year model.

|  | Population | | | Family | | |
| --- | --- | --- | --- | --- | --- | --- |
|  | χ^2^ | p | PVE | χ^2^ | p | PVE |
| Height before flowering | 0.000 | 0.500 | 0.000 | 1.477 | 0.112 | 3.438 |
| Height after flowering | 0.776 | 0.189 | 1.442 | 5.626 | **0.009** | 6.616 |
| LDMC | 0.000 | 0.380 | 0.407 | 0.000 | 0.500 | 0.000 |
| Mortality | 0.795 | 0.186 | 1.410 | 0.778 | 0.189 | 2.746 |
| Ramets before flowering | 0.032 | 0.429 | 0.000 | 68.887 | **<0.001** | 6.927 |
| Ramets after flowering | 0.121 | 0.364 | 0.324 | 27.650 | **<0.001** | 5.643 |
| Relative growth rate | 0.000 | 0.500 | 0.000 | 0.073 | 0.394 | 1.051 |
| SLA | 0.059 | 0.404 | 0.349 | 0.000 | 0.500 | 0.000 |
| Monarch butterfly abundance | 0.070 | 0.396 | 0.354 | 4.087 | **0.021** | 0.000 |
| Swamp milkweed beetle abundance | 0.000 | 0.500 | 0.675 | 0.000 | 0.500 | 0.000 |
| Milkweed leaf-mining fly abundance | 1.555 | 0.106 | 2.159 | 1.144 | 0.142 | 2.320 |
| Herbivory before flowering (binary) | 0.000 | 0.500 | 0.000 | 0.001 | 0.490 | 0.317 |
| Herbivory before flowering (quantitative) | 0.000 | 0.500 | 0.000 | 0.000 | 0.500 | 0.000 |
| Herbivory after flowering (binary) | 0.000 | 0.496 | 0.000 | 7.092 | **0.004** | 1.948 |
| Herbivory after flowering (quantitative) | 0.000 | 0.500 | 0.000 | 0.413 | 0.260 | 2.163 |
| Latex exudation | 4.536 | **0.016** | 4.045 | 2.033 | 0.077 | 4.748 |
| Milkweed stem weevil damage (binary) | 0.000 | 0.500 | 0.000 | 3.501 | **0.030** | 5.624 |
| Milkweed stem weevil damage (quantitative) | 1.208 | 0.136 | 2.112 | 1.189 | 0.138 | 4.006 |
| Date of first flower | 0.000 | 0.500 | 0.000 | 84.743 | **<0.001** | 9.564 |
| Date of first follicle | 0.000 | 0.500 | 0.000 | 47.484 | **<0.001** | 0.000 |
| Flower size | 1.223 | 0.134 | 7.189 | 0.110 | 0.370 | 4.096 |
| Flowering duration | 0.000 | 0.500 | 0.000 | 0.000 | 0.500 | 0.000 |
| Flowering success | 4.855 | **0.014** | 3.420 | 0.267 | 0.302 | 1.443 |
| Follicles | 0.000 | 0.500 | 0.000 | 0.000 | 0.500 | 0.000 |
| Inflorescences | 3.285 | **0.035** | 7.409 | 0.000 | 0.500 | 0.000 |
| Flowers per inflorescence | 0.833 | 0.180 | 8.369 | 0.000 | 0.500 | 0.000 |
| Pollinaria removed | 0.017 | 0.449 | 1.003 | 3.304 | **0.034** | 27.192 |

**Supplementary Table 4.** Results from general and generalized linear mixed effect models examining the amount of heritable genetic variation within and among populations. All populations were included. Maximum likelihood χ^2^ and p-values were obtained from type III sums-of-squares ANOVA performed on random effects.

|  | Distance | | | | | | Urbanization Score | | | | | |
| --- | --- | --- | --- | --- | --- | --- | --- | --- | --- | --- | --- | --- |
|  | Population | | | Family | | | Population | | | Family | | |
|  | χ^2^ | p | PVE | χ^2^ | p | PVE | χ^2^ | p | PVE | χ^2^ | p | PVE |
| Height before flowering | 0.000 | 0.500 | 0.000 | 1.485 | 0.112 | 3.454 | 0.000 | 0.500 | 0.000 | 1.565 | 0.106 | 3.552 |
| Height after flowering | 0.680 | 0.205 | 1.349 | 5.698 | **0.009** | 6.666 | 0.873 | 0.175 | 1.547 | 5.642 | **0.009** | 6.618 |
| LDMC | 0.000 | 0.424 | 0.254 | 0.000 | 0.500 | 0.000 | 0.157 | 0.346 | 0.537 | 0.000 | 0.500 | 0.000 |
| Mortality | 0.804 | 0.185 | 1.571 | 0.781 | 0.188 | 2.738 | 0.820 | 0.182 | 1.586 | 0.758 | 0.192 | 2.701 |
| Ramets before flowering | 0.001 | 0.485 | 0.000 | 68.905 | **<0.001** | 6.877 | 0.034 | 0.427 | 0.000 | 68.829 | **<0.001** | 7.049 |
| Ramets after flowering | 0.025 | 0.437 | 0.248 | 27.856 | **<0.001** | 5.669 | 0.114 | 0.368 | 0.446 | 27.672 | **<0.001** | 5.650 |
| Relative growth rate | 0.000 | 0.500 | 0.000 | 0.098 | 0.377 | 1.229 | 0.000 | 0.500 | 0.000 | 0.101 | 0.376 | 1.243 |
| SLA | 0.126 | 0.362 | 0.521 | 0.000 | 0.500 | 0.000 | 0.100 | 0.376 | 0.463 | 0.000 | 0.500 | 0.000 |
| Monarch butterfly abundance | 0.004 | 0.476 | 0.209 | 4.198 | **0.020** | 0.004 | 0.000 | 0.500 | 0.056 | 4.317 | **0.019** | 0.084 |
| Swamp milkweed beetle abundance | 0.000 | 0.500 | 0.772 | 0.000 | 0.500 | 0.000 | 0.000 | 0.500 | 0.730 | 0.000 | 0.500 | 0.000 |
| Milkweed leaf-mining fly abundance | 1.538 | 0.108 | 2.304 | 1.168 | 0.140 | 2.322 | 1.295 | 0.128 | 2.201 | 1.200 | 0.136 | 2.318 |
| Herbivory before flowering (binary) | 0.000 | 0.500 | 0.000 | 0.000 | 0.500 | 0.000 | 0.000 | 0.500 | 0.000 | 0.000 | 0.500 | 0.331 |
| Herbivory before flowering (quantitative) | 0.000 | 0.500 | 0.000 | 0.000 | 0.500 | 0.000 | 0.000 | 0.500 | 0.000 | 0.000 | 0.500 | 0.000 |
| Herbivory after flowering (binary) | 0.000 | 0.494 | 0.000 | 5.815 | **0.008** | 1.937 | 0.001 | 0.486 | 0.000 | 5.699 | **0.009** | 1.894 |
| Herbivory after flowering (quantitative) | 0.000 | 0.500 | 0.000 | 0.495 | 0.241 | 2.370 | 0.000 | 0.500 | 0.000 | 0.482 | 0.244 | 2.348 |
| Latex exudation | 4.628 | **0.016** | 4.141 | 2.052 | 0.076 | 4.770 | 3.010 | **0.042** | 3.217 | 1.907 | 0.084 | 4.631 |
| Milkweed stem weevil damage (binary) | 0.000 | 0.500 | 0.000 | 3.429 | **0.032** | 5.637 | 0.000 | 0.500 | 0.000 | 3.499 | **0.030** | 5.751 |
| Milkweed stem weevil damage (quantitative) | 1.232 | 0.134 | 2.128 | 1.049 | 0.153 | 3.755 | 1.392 | 0.119 | 2.291 | 1.095 | 0.148 | 3.839 |
| Date of first flower | 0.000 | 0.500 | 0.000 | 84.846 | **<0.001** | 10.098 | 0.000 | 0.500 | 0.000 | 85.034 | **<0.001** | 9.954 |
| Date of first follicle | 0.003 | 0.476 | 0.000 | 45.814 | **<0.001** | 0.000 | 0.000 | 0.500 | 0.000 | 47.014 | **<0.001** | 0.000 |
| Flower size | 1.159 | 0.141 | 7.233 | 0.103 | 0.374 | 3.994 | 0.833 | 0.181 | 6.365 | 0.132 | 0.358 | 4.603 |
| Flowering duration | 0.000 | 0.500 | 0.000 | 0.000 | 0.500 | 0.000 | 0.000 | 0.500 | 0.000 | 0.000 | 0.500 | 0.000 |
| Flowering success | 4.766 | **0.015** | 3.593 | 0.268 | 0.302 | 1.439 | 4.695 | **0.015** | 3.569 | 0.274 | 0.300 | 1.453 |
| Follicles | 0.000 | 0.500 | 0.743 | 0.000 | 0.500 | 0.000 | 0.000 | 0.500 | 0.813 | 0.000 | 0.500 | 0.000 |
| Inflorescences | 3.283 | **0.035** | 8.156 | 0.000 | 0.500 | 0.000 | 3.070 | **0.040** | 7.853 | 0.000 | 0.500 | 0.000 |
| Flowers per inflorescence | 0.203 | 0.326 | 6.489 | 0.000 | 0.500 | 1.507 | 0.060 | 0.403 | 6.237 | 0.000 | 0.500 | 0.838 |
| Pollinaria removed | 0.103 | 0.374 | 2.328 | 2.918 | **0.044** | 25.028 | 0.088 | 0.384 | 2.174 | 2.722 | **0.050** | 24.373 |

**Supplementary Table 5.** Results from general and generalized linear mixed effect models examining the amount of heritable genetic variation associated with urbanization within and among populations. All populations were included. Maximum likelihood χ^2^ and p-values were obtained from type III sums-of-squares ANOVA performed on random effects.

| Variable | Predictor | SS | df | F | *p* |
| --- | --- | --- | --- | --- | --- |
| Total Cardenolides | Distance to City Center | 0.049 | 1, 49 | 3.210 | 0.079 |
|  | Urbanization Score | 0.002 | 1, 49 | 0.145 | 0.705 |
| Glycosylated Aspecioside | Distance to City Center | 0.037 | 1, 49 | 3.463 | 0.069 |
|  | Urbanization Score | 0.002 | 1, 49 | 0.144 | 0.706 |
| Labriformin | Distance to City Center | 0.001 | 1, 49 | 0.482 | 0.491 |
|  | Urbanization Score | 0.000 | 1, 49 | 0.385 | 0.538 |
| Cardenolide 17.6 | Distance to City Center | 0.000 | 1, 49 | 0.353 | 0.555 |
|  | Urbanization Score | 1.254 | 1, 49 | 3.480 | 0.068 |

**Supplementary Table 6.** Results from general linear models examining the effects of urbanization on cardenolide concentration. All populations were included. Shown are sums of squares (SS), degrees of freedom (df), F statistics, and p-values obtained from type III ANOVA. Cardenolide 17.6 is an unidentified cardenolide with a retention time of 17.6 minutes.

|  | | **Distance to City Center** | | | | **Urbanization Score** | | | |
| --- | --- | --- | --- | --- | --- | --- | --- | --- | --- |
|  | | **All Populations** | | **Urban Populations** | | **All Populations** | | **Urban Populations** | |
|  | | **Model 1** | | **Model 2** | | **Model 3** | | **Model 4** | |
| **Trait** | **Pseudo-R^2^ Method** | **R^2^m** | **R^2^c** | **R^2^m** | **R^2^c** | **R^2^m** | **R^2^c** | **R^2^m** | **R^2^c** |
| Latex exudation | - | 0.037 | 0.119 | 0.043 | 0.145 | 0.045 | 0.118 | 0.060 | 0.141 |
| Herbivory before flowering (binary) | delta | 0.015 | 0.015 | 0.013 | 0.013 | 0.011 | 0.014 | 0.016 | 0.016 |
| Herbivory before flowering (quantitative) | - | 0.010 | 0.010 | 0.001 | 0.001 | 0.016 | 0.016 | 0.002 | 0.002 |
| Herbivory after flowering (binary) | delta | 0.018 | 0.035 | 0.043 | 0.070 | 0.018 | 0.034 | 0.041 | 0.067 |
| Herbivory after flowering (quantitative) | - | 0.007 | 0.030 | 0.009 | 0.050 | 0.005 | 0.029 | 0.018 | 0.054 |
| Milkweed stem weevil damage (binary) | delta | 0.027 | 0.077 | 0.031 | 0.090 | 0.026 | 0.078 | 0.034 | 0.093 |
| Milkweed stem weevil damage (quantitative) | - | 0.023 | 0.081 | 0.018 | 0.141 | 0.021 | 0.081 | 0.018 | 0.141 |
| Flowering success | delta | 0.038 | 0.088 | 0.037 | 0.109 | 0.038 | 0.088 | 0.042 | 0.114 |
| Flowers per inflorescence | trigamma | 0.000 | 0.001 | 0.000 | 0.001 | 0.001 | 0.001 | 0.000 | 0.001 |
| Flower size | - | 0.019 | 0.129 | 0.034 | 0.197 | 0.021 | 0.128 | 0.030 | 0.208 |
| Flowering duration | trigamma | 0.000 | 0.000 | 0.001 | 0.002 | 0.001 | 0.001 | 0.003 | 0.003 |
| Date of first flower | trigamma | 0.000 | 0.000 | 0.000 | 0.000 | 0.000 | 0.000 | 0.000 | 0.000 |
| Pollinaria removed | - | 0.133 | 0.370 | 0.097 | 0.245 | 0.130 | 0.361 | 0.103 | 0.227 |
| Follicles | trigamma | 0.161 | 0.161 | 0.243 | 0.322 | 0.160 | 0.160 | 0.325 | 0.325 |
| Date of first follicle | trigamma | 0.055 | 0.690 | 0.159 | 0.678 | 0.053 | 0.691 | 0.167 | 0.679 |
| Inflorescences | trigamma | 0.000 | 0.000 | 0.000 | 0.000 | 0.000 | 0.000 | 0.000 | 0.000 |
| Monarch butterfly abundance | trigamma | 0.013 | 0.029 | 0.013 | 0.029 | 0.011 | 0.028 | 0.011 | 0.028 |
| Milkweed leaf-mining fly abundance | trigamma | 0.000 | 0.000 | 0.000 | 0.000 | 0.000 | 0.000 | 0.001 | 0.008 |
| Swamp milkweed beetle abundance | trigamma | 0.000 | 0.000 | 0.054 | 0.345 | 0.000 | 0.000 | 0.000 | 0.000 |
| LDMC | - | 0.058 | 0.058 | 0.007 | 0.022 | 0.056 | 0.056 | 0.059 | 0.072 |
| SLA | - | 0.065 | 0.071 | 0.068 | 0.108 | 0.065 | 0.071 | 0.070 | 0.106 |
| Height before flowering | - | 0.076 | 0.108 | 0.075 | 0.115 | 0.075 | 0.108 | 0.075 | 0.114 |
| Height after flowering | - | 0.081 | 0.154 | 0.086 | 0.169 | 0.079 | 0.154 | 0.074 | 0.164 |
| Relative growth rate | - | 0.014 | 0.026 | 0.021 | 0.031 | 0.014 | 0.027 | 0.021 | 0.030 |
| Ramets before flowering | trigamma | 0.080 | 0.269 | 0.094 | 0.297 | 0.078 | 0.270 | 0.081 | 0.290 |
| Ramets after flowering | trigamma | 0.102 | 0.204 | 0.121 | 0.224 | 0.100 | 0.204 | 0.111 | 0.217 |
| Mortality | delta | 0.035 | 0.075 | 0.040 | 0.123 | 0.035 | 0.075 | 0.039 | 0.122 |

**Supplementary Table 7.** Marginal and conditional R^2^ values from general and generalized linear mixed effect models examining the effects of urbanization and a green corridor on all phenotypic traits. Shown are traits, method for deriving the observation-level variance for generalized linear mixed effect models when calculated as pseudo-R², and marginal and conditional R² values when urbanization was quantified by distance from the urban center (Models 1-2) and urbanization score (Models 3-4), and when all populations were included (Models 1 & 3) and only urban sites were included (Models 2 & 4).

| **Variable** | **Urbanization** | **R^2^** | **R^2^_adj_** |
| --- | --- | --- | --- |
| Total Cardenolides | Distance to City Center | 0.061 | 0.042 |
|  | Urbanization Score | 0.003 | -0.017 |
| Glycosylated Aspecioside | Distance to City Center | 0.066 | 0.047 |
|  | Urbanization Score | 0.003 | -0.017 |
| Labriformin | Distance to City Center | 0.010 | -0.010 |
|  | Urbanization Score | 0.008 | -0.012 |
| Cardenolide 17.6 | Distance to City Center | 0.007 | -0.013 |
|  | Urbanization Score | 0.066 | 0.047 |

**Supplementary Table 8.** Standard and adjusted R² values from general linear models examining the effects of urbanization on cardenolide concentrations. All populations were included. Cardenolide 17.6 is an unidentified cardenolide with a retention time of 17.6 minutes.

| Variable | Original p | Benjamini-Hochberg |
| --- | --- | --- |
| Herbivory before flowering (quantitative) | 0.058 | 0.527 |
| Pollinaria removed | 0.058 | 0.527 |
| Glycosylated Aspecioside | 0.069 | 0.527 |
| Total cardenolides | 0.079 | 0.527 |
| Herbivory before flowering (binary) | 0.085 | 0.527 |
| Flowers per inflorescence | 0.147 | 0.589 |
| LDMC | 0.162 | 0.589 |
| Ramets before flowering | 0.170 | 0.589 |
| Ramets after flowering | 0.171 | 0.589 |
| Weevil damage (quantitative) | 0.204 | 0.603 |
| Monarch butterfly abundance | 0.214 | 0.603 |
| Height after flowering | 0.249 | 0.618 |
| Height before flowering | 0.259 | 0.618 |
| Herbivory after flowering (quantitative) | 0.307 | 0.680 |
| Herbivory after flowering (binary) | 0.356 | 0.736 |
| Weevil damage (binary) | 0.398 | 0.771 |
| Flower size | 0.438 | 0.799 |
| Labriformin | 0.491 | 0.845 |
| Date of first follicle | 0.545 | 0.861 |
| Cardenolide 17.6 | 0.555 | 0.861 |
| Latex exudation | 0.620 | 0.915 |
| Date of first flower | 0.733 | 0.945 |
| Mortality | 0.747 | 0.945 |
| SLA | 0.752 | 0.945 |
| Milkweed leaf-mining fly abundance | 0.762 | 0.945 |
| Swamp milkweed leaf beetle abundance | 0.848 | 0.990 |
| Inflorescences | 0.899 | 0.990 |
| Follicles | 0.906 | 0.990 |
| Flowering duration | 0.937 | 0.990 |
| Flowering success | 0.978 | 0.990 |
| Relative growth rate | 0.990 | 0.990 |

**Supplementary Table 9.** Results from false discovery rate tests of general and generalized linear mixed effect models examining the effects of urbanization on all phenotypic traits. Urbanization was quantified via distance from the urban center and all populations were included. Shown are original p-values obtained from type III sums-of-squares ANOVA and adjusted values using the Benjamini-Hochberg procedure.

| Variable | Original p | Benjamini-Hochberg |
| --- | --- | --- |
| Herbivory before flowering (quantitative) | 0.013 | 0.403 |
| Latex exudation | 0.045 | 0.434 |
| Flowers per inflorescence | 0.059 | 0.434 |
| Cardenolide 17.6 | 0.068 | 0.434 |
| Pollinaria removed | 0.070 | 0.434 |
| Monarch butterfly abundance | 0.159 | 0.822 |
| Flower size | 0.373 | 0.917 |
| Milkweed leaf-mining fly abundance | 0.414 | 0.917 |
| Herbivory after flowering (binary) | 0.433 | 0.917 |
| Weevil damage (quantitative) | 0.447 | 0.917 |
| Herbivory before flowering (binary) | 0.508 | 0.917 |
| Date of first flower | 0.519 | 0.917 |
| Labriformin | 0.538 | 0.917 |
| Height after flowering | 0.550 | 0.917 |
| Height before flowering | 0.581 | 0.917 |
| LDMC | 0.609 | 0.917 |
| Inflorescences | 0.614 | 0.917 |
| Mortality | 0.652 | 0.917 |
| Swamp milkweed leaf beetle abundance | 0.656 | 0.917 |
| Relative growth rate | 0.697 | 0.917 |
| Total cardenolides | 0.705 | 0.917 |
| Glycosylated Aspecioside | 0.706 | 0.917 |
| SLA | 0.714 | 0.917 |
| Follicles | 0.767 | 0.917 |
| Ramets before flowering | 0.777 | 0.917 |
| Ramets after flowering | 0.820 | 0.917 |
| Weevil damage (binary) | 0.832 | 0.917 |
| Flowering duration | 0.853 | 0.917 |
| Flowering success | 0.876 | 0.917 |
| Herbivory after flowering (quantitative) | 0.887 | 0.917 |
| Date of first follicle | 0.981 | 0.981 |

**Supplementary Table 10.** Results from false discovery rate tests of general and generalized linear mixed effect models examining the effects of urbanization on all phenotypic traits. Urbanization was quantified via urbanization score and all populations were included. Shown are original p-values obtained from type III sums-of-squares ANOVA and adjusted values using the Benjamini-Hochberg procedure.

|  | Distance | | | | | | Urbanization Score | | | | | |
| --- | --- | --- | --- | --- | --- | --- | --- | --- | --- | --- | --- | --- |
|  | Population | | | Family | | | Population | | | Family | | |
|  | χ^2^ | p | PVE | χ^2^ | p | PVE | χ^2^ | p | PVE | χ^2^ | p | PVE |
| Height before flowering | 0.000 | 0.500 | 0.000 | 1.681 | 0.098 | 4.617 | 0.000 | 0.500 | 0.000 | 1.575 | 0.104 | 4.475 |
| Height after flowering | 0.000 | 0.500 | 0.000 | 7.154 | **0.004** | 9.027 | 0.050 | 0.411 | 0.485 | 7.029 | **0.004** | 9.265 |
| LDMC | 0.050 | 0.412 | 0.413 | 0.011 | 0.459 | 0.439 | 0.211 | 0.323 | 0.888 | 0.013 | 0.454 | 0.492 |
| Mortality | 0.000 | 0.500 | 1.067 | 3.952 | **0.024** | 7.163 | 0.000 | 0.500 | 0.923 | 3.965 | **0.023** | 7.079 |
| Ramets before flowering | 0.000 | 0.500 | 0.165 | 52.455 | **<0.001** | 7.884 | 0.000 | 0.500 | 0.473 | 52.334 | **<0.001** | 7.827 |
| Ramets after flowering | 0.000 | 0.500 | 0.000 | 22.590 | **<0.001** | 5.826 | 0.000 | 0.500 | 0.000 | 22.903 | **<0.001** | 5.999 |
| Relative growth rate | 0.483 | 0.244 | 1.389 | 0.000 | 0.500 | 0.000 | 0.237 | 0.314 | 0.973 | 0.000 | 0.500 | 0.000 |
| SLA | 2.440 | 0.059 | 3.728 | 0.037 | 0.424 | 0.785 | 2.258 | 0.066 | 3.486 | 0.006 | 0.469 | 0.319 |
| Monarch butterfly abundance | 0.000 | 0.500 | 0.000 | 0.000 | 0.500 | 0.000 | 0.017 | 0.448 | 0.437 | 0.044 | 0.418 | 0.000 |
| Swamp milkweed leaf beetle abundance | 0.000 | 0.500 | 0.800 | 0.000 | 0.500 | 0.000 | 0.000 | 0.500 | 0.000 | 0.000 | 0.500 | 0.000 |
| Milkweed leaf-mining fly abundance | 0.034 | 0.428 | 1.673 | 3.365 | **0.034** | 4.881 | 0.055 | 0.407 | 1.583 | 3.181 | **0.038** | 4.849 |
| Herbivory before flowering (binary) | 0.000 | 0.500 | 0.000 | 0.000 | 0.500 | 0.000 | 0.000 | 0.500 | 0.000 | 0.000 | 0.500 | 0.000 |
| Herbivory before flowering (quantitative) | 0.000 | 0.500 | 0.000 | 0.000 | 0.500 | 0.000 | 0.000 | 0.500 | 0.000 | 0.000 | 0.500 | 0.000 |
| Herbivory after flowering (binary) | 0.001 | 0.490 | 0.000 | 0.315 | 0.288 | 3.729 | 0.001 | 0.488 | 0.000 | 0.280 | 0.298 | 3.809 |
| Herbivory after flowering (quantitative) | 0.000 | 0.500 | 0.000 | 1.076 | 0.150 | 4.410 | 0.000 | 0.500 | 0.000 | 0.743 | 0.194 | 3.628 |
| Latex exudation | 2.000 | 0.055 | 3.946 | 3.074 | **0.040** | 7.114 | 0.504 | 0.239 | 1.722 | 3.228 | **0.036** | 7.465 |
| Milkweed stem weevil damage (binary) | 0.000 | 0.500 | 0.000 | 2.741 | **0.049** | 6.715 | 0.000 | 0.500 | 0.000 | 2.614 | 0.053 | 6.614 |
| Milkweed stem weevil damage (quantitative) | 2.144 | 0.072 | 4.356 | 3.595 | **0.029** | 8.479 | 1.603 | 0.102 | 3.912 | 3.715 | **0.027** | 8.672 |
| Date of first flower | 0.000 | 0.500 | 0.000 | 52.522 | **<0.001** | 12.682 | 0.000 | 0.500 | 0.000 | 53.835 | **<0.001** | 10.791 |
| Date of first follicle | 0.000 | 0.500 | 0.000 | 29.669 | **<0.001** | 0.000 | 0.000 | 0.500 | 0.000 | 30.383 | **<0.001** | 0.000 |
| Flower size | 1.331 | 0.124 | 10.569 | 0.319 | 0.286 | 8.428 | 0.845 | 0.179 | 9.030 | 0.414 | 0.260 | 9.935 |
| Flowering duration | 0.000 | 0.500 | 0.769 | 0.000 | 0.500 | 0.000 | 0.000 | 0.500 | 0.000 | 0.000 | 0.500 | 0.000 |
| Flowering success | 0.115 | 0.368 | 2.045 | 2.696 | 0.051 | 5.187 | 0.025 | 0.437 | 1.459 | 2.814 | **0.046** | 5.308 |
| Follicles | 0.000 | 0.500 | 14.430 | 0.000 | 0.500 | 11.954 | 0.000 | 0.500 | 5.764 | 0.000 | 0.500 | 11.434 |
| Inflorescences | 1.325 | 0.125 | 12.878 | 9.892 | **0.001** | 0.000 | 0.930 | 0.168 | 7.011 | 9.238 | **0.001** | 0.000 |
| Flowers per inflorescence | 0.359 | 0.274 | 11.915 | 0.000 | 0.500 | 1.850 | 0.000 | 0.500 | 9.792 | 0.000 | 0.500 | 1.128 |
| Pollinaria removed | 0.145 | 0.352 | 3.139 | 0.628 | 0.214 | 14.544 | 0.035 | 0.426 | 1.477 | 0.345 | 0.278 | 11.331 |

**Supplementary Table 11.** Results from general and generalized linear mixed effect models examining the amount of heritable genetic variation associated with urbanization and proximity to a green corridor within and among populations. Only urban populations were included. Maximum likelihood χ^2^ and p-values were obtained from type III sums-of-squares ANOVA performed on random effects.

|  | Urbanization Score | | Subtransect | | U x S | | Individuals | Populations |
| --- | --- | --- | --- | --- | --- | --- | --- | --- |
|  | χ^2^ | p | χ^2^ | p | χ^2^ | p |  |  |
| Latex exudation | 9.011 | **0.003** | 4.840 |  |  | **0.023** | 474 | 35 |
| Herbivory before flowering (binary) | 2.286 | 0.131 | 0.860 | 0.354 |  |  | 534 | 35 |
| Herbivory before flowering (quantitative) | 0.298 | 0.585 | 0.023 | 0.878 |  |  | 303 | 35 |
| Herbivory after flowering (binary) | 0.104 | 0.747 | 0.600 | 0.439 |  |  | 466 | 35 |
| Herbivory after flowering (quantitative) | 1.457 | 0.227 | 1.155 | 0.282 | 3.528 | 0.060 | 450 | 35 |
| Milkweed stem weevil damage (binary) | 2.701 | 0.100 | 2.684 | 0.101 | 4.667 | **0.031** | 635 | 36 |
| Milkweed stem weevil damage (quantitative) | 0.017 | 0.896 | 0.806 | 0.369 |  |  | 464 | 35 |
| Flowering success | 0.391 | 0.532 | 0.435 | 0.509 | 2.088 | 0.148 | 634 | 36 |
| Flowers per inflorescence | 2.158 | 0.142 | 5.572 | **0.018** |  |  | 126 | 33 |
| Flower size | 1.066 | 0.302 | 0.292 | 0.589 |  |  | 124 | 33 |
| Flowering duration | 5.274 | **0.022** | 2.108 | 0.147 | 7.614 | **0.006** | 126 | 33 |
| Date of first flower | 0.092 | 0.762 | 0.003 | 0.956 |  |  | 126 | 33 |
| Pollinaria removed | 1.336 | 0.248 | 0.173 | 0.677 |  |  | 124 | 33 |
| Follicles | 5.185 | **0.023** | 6.494 | **0.011** | 7.454 | **0.006** | 126 | 33 |
| Date of first follicle | 2.108 | 0.147 | 1.113 | 0.291 | 4.467 | **0.035** | 90 | 30 |
| Inflorescences | 4.233 | **0.040** | 2.101 | 0.147 | 5.252 | **0.022** | 125 | 33 |
| Monarch butterfly abundance | 0.072 | 0.788 | 0.019 | 0.889 | 2.213 | 0.137 | 635 | 36 |
| Milkweed leaf-mining fly abundance | 0.175 | 0.676 | 0.150 | 0.698 |  |  | 635 | 36 |
| Swamp milkweed leaf beetle abundance | 0.193 | 0.661 | 0.600 | 0.439 |  |  | 635 | 36 |
| LDMC | 0.720 | 0.396 | 0.646 | 0.421 |  |  | 533 | 35 |
| SLA | 1.260 | 0.262 | 1.438 | 0.230 |  |  | 516 | 35 |
| Height before flowering | 0.275 | 0.600 | 0.003 | 0.954 |  |  | 555 | 35 |
| Height after flowering | 0.025 | 0.875 | 0.227 | 0.634 |  |  | 635 | 36 |
| Relative growth rate | 0.113 | 0.737 | 0.431 | 0.512 |  |  | 392 | 35 |
| Ramets before flowering | 0.761 | 0.383 | 0.161 | 0.688 |  |  | 635 | 36 |
| Ramets after flowering | 1.499 | 0.221 | 1.263 | 0.261 |  |  | 635 | 36 |
| Mortality | 0.332 | 0.564 | 0.063 | 0.802 |  |  | 634 | 36 |

**Supplementary Table 12.** Results from general and generalized linear mixed effect models examining the effects of urbanization and proximity to a green corridor on all phenotypic traits. Urbanization was quantified via urbanization score and only urban populations were included. Shown are maximum likelihood χ^2^ and p-values obtained from type III sums-of-squares ANOVA. Though not shown, block was included as a fixed effect and often explained significant variation in the common garden.

| Variable | Original p | Benjamini-Hochberg |
| --- | --- | --- |
| Flowers per inflorescence | 0.039 | 0.837 |
| Ramets after flowering | 0.072 | 0.837 |
| Height after flowering | 0.124 | 0.837 |
| Ramets before flowering | 0.152 | 0.837 |
| Flowering duration | 0.155 | 0.837 |
| LDMC | 0.228 | 0.845 |
| Date of first follicle | 0.331 | 0.845 |
| SLA | 0.360 | 0.845 |
| Weevil damage (quantitative) | 0.363 | 0.845 |
| Herbivory after flowering (binary) | 0.412 | 0.845 |
| Flowering success | 0.429 | 0.845 |
| Follicles | 0.446 | 0.845 |
| Flower size | 0.448 | 0.845 |
| Relative growth rate | 0.453 | 0.845 |
| Herbivory before flowering (binary) | 0.497 | 0.845 |
| Inflorescences | 0.501 | 0.845 |
| Herbivory after flowering (quantitative) | 0.533 | 0.847 |
| Milkweed leaf-mining fly abundance | 0.681 | 0.933 |
| Weevil damage (binary) | 0.684 | 0.933 |
| Monarch butterfly abundance | 0.691 | 0.933 |
| Herbivory before flowering (quantitative) | 0.727 | 0.935 |
| Swamp milkweed leaf beetle abundance | 0.794 | 0.939 |
| Mortality | 0.803 | 0.939 |
| Latex exudation | 0.906 | 0.939 |
| Pollinaria removed | 0.912 | 0.939 |
| Height before flowering | 0.925 | 0.939 |
| Date of first flower | 0.939 | 0.939 |

**Supplementary Table 13.** Results from false discovery rate tests of general and generalized linear mixed effect models examining the effects of urbanization and proximity to a green corridor on all phenotypic traits. Urbanization was quantified via distance from the urban center and only urban populations were included. Shown are original p-values obtained from type III sums-of-squares ANOVA and adjusted values using the Benjamini-Hochberg procedure.

| Variable | Original p | Benjamini-Hochberg |
| --- | --- | --- |
| Flowering duration | 0.006 | 0.081 |
| Follicles | 0.006 | 0.081 |
| Flowers per inflorescence | 0.018 | 0.124 |
| Inflorescences | 0.022 | 0.124 |
| Latex exudation | 0.023 | 0.124 |
| Weevil damage (binary) | 0.031 | 0.135 |
| Date of first follicle | 0.035 | 0.135 |
| Herbivory after flowering (quantitative) | 0.060 | 0.202 |
| Monarch butterfly abundance | 0.137 | 0.400 |
| Flowering success | 0.148 | 0.400 |
| SLA | 0.230 | 0.565 |
| Ramets after flowering | 0.261 | 0.587 |
| Herbivory before flowering (binary) | 0.354 | 0.697 |
| Weevil damage (quantitative) | 0.369 | 0.697 |
| LDMC | 0.421 | 0.697 |
| Herbivory after flowering (binary) | 0.439 | 0.697 |
| Swamp milkweed leaf beetle abundance | 0.439 | 0.697 |
| Relative growth rate | 0.512 | 0.768 |
| Flower size | 0.589 | 0.819 |
| Height after flowering | 0.634 | 0.819 |
| Pollinaria removed | 0.677 | 0.819 |
| Ramets before flowering | 0.688 | 0.819 |
| Milkweed leaf-mining fly abundance | 0.698 | 0.819 |
| Mortality | 0.802 | 0.902 |
| Herbivory before flowering (quantitative) | 0.878 | 0.948 |
| Height before flowering | 0.954 | 0.956 |
| Date of first flower | 0.956 | 0.956 |

**Supplementary Table 14.** Results from false discovery rate tests of general and generalized linear mixed effect models examining the effects of urbanization and proximity to a green corridor on all phenotypic traits. Urbanization was quantified via urbanization score and only urban populations were included. Shown are original p-values obtained from type III sums-of-squares ANOVA and adjusted values using the Benjamini-Hochberg procedure.

## **References**

1. Schneider, C. A., Rasband, W. S. & Eliceiri, K. W. NIH Image to ImageJ: 25 years of image analysis. *Nat. Methods* **9**, 671–675 (2012).
